# Functional characterization of *Schistosoma mansoni* fucosyltransferases in *Nicotiana benthamiana* plants

**DOI:** 10.1038/s41598-020-74485-z

**Published:** 2020-10-28

**Authors:** Kim van Noort, Dieu-Linh Nguyen, Verena Kriechbaumer, Chris Hawes, Cornelis H. Hokke, Arjen Schots, Ruud H. P. Wilbers

**Affiliations:** 1 Laboratory of Nematology, Plant Sciences Group, Wageningen University and Research, Droevendaalsesteeg 1, 6708 PB Wageningen, The Netherlands; 2 Department of Parasitology, Leiden University Medical Center, Albinusdreef, 2333 ZA Leiden, The Netherlands; 3 Department of Biological and Medical Sciences, Oxford Brookes University, Oxford, OX3 0BP UK

**Keywords:** Immune evasion, Vaccines, Biotechnology, Expression systems, Molecular engineering, Plant biotechnology

## Abstract

Helminth parasites secrete a wide variety of immunomodulatory proteins and lipids to dampen host immune responses. Many of these immunomodulatory compounds are modified with complex sugar structures (or glycans), which play an important role at the host–parasite interface. As an example, the human blood fluke *Schistosoma mansoni* produces highly fucosylated glycan structures on glycoproteins and glycolipids. Up to 20 different *S. mansoni* fucosyltransferase (SmFucT) genes can be found in genome databases, but thus far only one enzyme has been functionally characterized. To unravel the synthesis of highly fucosylated N-glycans by *S. mansoni*, we examined the ability of ten selected SmFucTs to modify N-glycans upon transient expression in *Nicotiana benthamiana* plants. All enzymes were localized in the plant Golgi apparatus, which allowed us to identify the SmFucTs involved in core fucosylation and the synthesis of complex antennary glycan motifs. This knowledge provides a starting point for investigations into the role of specific fucosylated glycan motifs of schistosomes in parasite-host interactions. The functionally characterized SmFucTs can also be applied to synthesize complex N-glycan structures on recombinant proteins to study their contribution to immunomodulation. Furthermore, this plant expression system will fuel the development of helminth glycoproteins for pharmaceutical applications or novel anti-helminth vaccines.

## Introduction

One of the most important neglected tropical parasitic diseases is caused by human parasitic trematodes of the genus *Schistosoma*, which together infect 252 million people worldwide^1^. Schistosomes have a complex lifecycle involving a human and snail host. Throughout the lifecycle these parasites secrete glycoproteins and glycolipids that affect the host immune system, often in a glycan-dependent manner. Notable among the secretions of *S. mansoni* are highly fucosylated glycan motifs that are differentially expressed during the lifecycle, and on specific glycoproteins^2–4^. N-glycans on glycoproteins of *S. mansoni* can be fucosylated at either the core, the antennae or both. Core fucosylation is an α1,3- or α1,6-linked fucose to the innermost *N*-acetyl-glucosamine (GlcNAc) residue on the N-glycan. On the N-glycan antenna complex motifs with fucose can be found, such as Lewis X (LeX or Galβ1-4(Fucα1-3)GlcNAc) and LDN-F (GalNAcβ1-4(Fucα1-3)GlcNAc)^3,5^. Additionally, a striking double fucose motif (with Fucα1-2Fuc linkage) is present on the N-glycans of *Schistosoma mansoni* venom allergen-like 9 (SmVAL-9) and this motif is also abundantly found on O-glycans and glycolipids^3,6^.

Glycans play an important role at the host–parasite interface and several glycan motifs commonly found in schistosomes and nematodes have been implicated in immunomodulation^7–10^. A well-known example is the incorporation of phosphorylcholine on N-glycans in nematodes, a glycan modification that by itself can mimic the effects of the immunomodulatory glycoprotein ES-62^11^. Additionally, the monosaccharide fucose can be highly immunogenic and many antibodies in the human host are directed against fucose containing glycan motifs^12^. Fucose-containing glycan motifs, such as LeX and LDN-F, interact with innate immune cells via C-type lectin receptors and can influence the outcome of immunological responses^13–17^. For instance, the Th2 inducing capacity of the *S. mansoni* soluble egg antigen omega-1 is glycosylation dependent. LeX on the N-glycans of omega-1 facilitates binding to the mannose receptor and DC-SIGN on dendritic cells (DCs), which mediates internalization of omega-1, and primes DCs for a Th2 response via its RNase activity^18^. Moreover, the presence of terminal LeX motifs on omega-1 N-glycans enhances its Th2 inducing ability in mice in comparison to omega-1 with paucimannosidic N-glycans^19^. Altogether, this underlines the importance of glycans and their contribution to modulation of host immune responses and role in parasite biology.

To be able to further investigate the role of fucosylated glycan motifs on parasite-secreted proteins and their role in schistosome-host biology, it is crucial to know how these fucosylated motifs are synthesized. Fucosylated glycan motifs are synthesized by specific fucosyltransferases (FucTs), however, most of the *S. mansoni* FucTs (SmFucTs) are not yet functionally characterized. Via a homology-based genome-wide bioinformatics approach, 14 full-length SmFucT sequences were characterized in silico and were previously amplified from cDNA^20^. Of these 14 sequences, two have been classified as O-SmFucTs involved in transfer of fucose to a serine or threonine residue. The other 12 SmFucTs (A-F and H-M) are divided into two groups, based on the type of linkage formed upon fucose transfer (either α1,3 or α1,6). To date, only SmFucTF has been functionally characterized and was shown to synthesize LeX in chemoenzymatic assays with various glycan acceptors^21^.

However, functional characterization based on chemoenzymatic assays might not reflect the actual function of a glycosyltransferase, since the characterization is performed outside the context of a cell. Glycosylation is a tightly regulated step-by-step process that occurs in the endoplasmatic reticulum (ER) and Golgi apparatus. The activity of different glycosyltransferases can depend on the specific localization in the Golgi, the surrounding pH, the formation of dimers and/or the availability of glycan acceptors and substrates. Glycosyltransferase activity is therefore highly dependent on subcellular localization, which is nearly impossible to mimic in vitro. Therefore, a recombinant expression system that allows Golgi localization of parasite glycosyltransferases would be beneficial for functional characterization studies.

Plants can be a promising platform for the characterization of parasite glycosyltransferases, since modifications of the plant glycosylation pathway do not influence its growth or development^22^. Over the last two decades, a wide variety of glycosyltransferases of animal origin have been successfully expressed in plants to synthesize tailored N-glycans^23^. Additionally, plants are available wherein endogenous plant FucTs are down-regulated by RNA interference, so-called ΔXT/FT plants^24^, which allows characterization of parasite FucTs. And finally, we have recently used *Nicotiana benthamiana* to produce glycoproteins from *S. mansoni* while simultaneously engineering their native N-glycan composition^19^. In that study we produced the egg antigens omega-1 and kappa-5, which naturally carry core fucosylated N-glycans with antennary Lewis X or LDN(-F).

In this study, we establish *N. benthamiana* as a novel in vivo characterization platform for parasite glycosyltransferase function. We functionally characterized SmFucTs by transient co-expression with the carrier glycoproteins omega-1 and kappa-5 from *S. mansoni* and subsequently analyzed the resulting N-glycan composition. We reveal the biological function of five out of ten selected SmFucTs. Furthermore, we can attribute a novel biological function to SmFucTF, which allows for the synthesis of the Fucα1-3GalNAcβ1-4(Fucα1-3)GlcNAc (F-LDN-F) motif in our plant-based expression platform.

## Results

### *Schistosoma mansoni* fucosyltransferases localize within the plant Golgi

Prior to the functional characterization of our selected SmFucTs we investigated whether these enzymes could be expressed heterologously in plant leaves. Simultaneously, we investigated whether they localize properly within the Golgi. GFP-tagged SmFucTs were co-expressed with RFP-tagged sub-Golgi reference markers GnTI-RFP, XylT-RFP and ST-RFP in *N. benthamiana*. GnTI-RFP localizes more to the *cis*-Golgi in comparison to XylT-RFP and the *trans*-Golgi localizing ST-RFP. XylT-RFP localizes in between GnTI-RFP and ST-RFP. Therefore, GnTI-RFP, XylT-RFP and ST-RFP are referred to as the *cis*/medial-, medial- and *trans*-Golgi marker, respectively.

**Figure 1:**
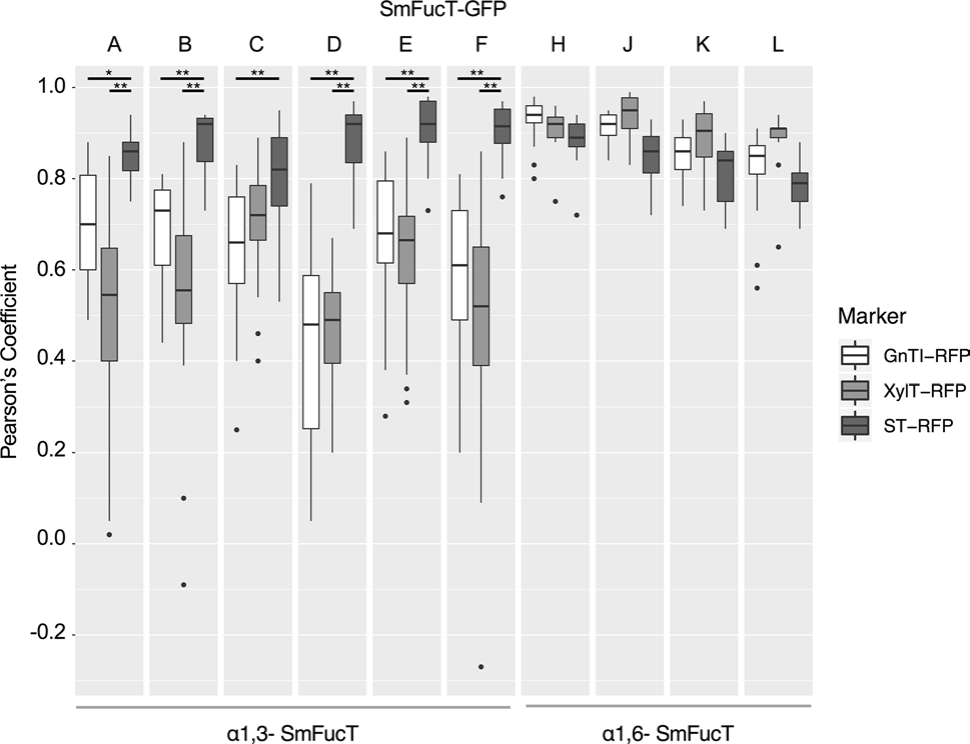
Golgi localization of *S. mansoni* fucosyltransferases (SmFucTs). GFP-tagged SmFucTs were co-expressed with the three different RFP-tagged Golgi reference markers (GnTI-RFP, XylT-RFP and ST-RFP) in *Nicotiana benthamiana* leaves (without P19). Co-localization analysis of side-on Golgi bodies (n ≥ 15) with the Pearson’s correlation coefficient of GFP-tagged SmFucTs co-expressed with the three RFP-tagged reference markers is presented as box plots. Significant differences according to a Tukey HSD test are indicated with asterisks (*P < 5.0 × 10^–3^; **P < 5.0 × 10^–7^).

In order to capture the fast-moving Golgi bodies, movies were made of which individual frames were analyzed. Golgi bodies tumble, therefore two different views can be distinguished, the ‘donut-shaped Golgi body’ and the ‘side-on view Golgi body’, which were both observed for all SmFucTs as illustrated in Figs. S1 (for SmFucTE) and S2. To determine the amount of co-localization between the SmFucTs and each individual reference marker, the Pearson’s correlation coefficient was determined using ‘side-on view Golgi bodies’ (Fig. 1, Fig. S1). Analysis of the Pearson’s correlation coefficients revealed that all ten SmFucTs showed a degree of co-localization with the *trans*-Golgi marker (ST-RFP), this indicates that all SmFucTs localized at least partly to the *trans*-Golgi. The degree of co-localization with the *trans*-Golgi marker significantly differed for SmFucTA, B, D, E and F in comparison to co-localization with the cis/medial- and medial-Golgi markers (GnTI-RFP and XylT-RFP, respectively). SmFucTC’s co-localization with the medial-Golgi marker was not significantly different from co-localization with the *cis*/medial- or *trans*-Golgi marker. This suggests that SmFucTC localized more towards the medial-Golgi than the other α1,3-SmFucTs. Co-localization studies of the α1,6-SmFucTs showed that no significant differences were observed for co-localization of SmFucTH and J to L with the three reference markers. This indicates that SmFucTH and J to L localized throughout the Golgi or the observed background noise hampered co-localization analysis. All-in-all these results reveal Golgi localization for each SmFucT in plant cells, which will allow functional characterization studies in *N. benthamiana*.

### Core fucosylation by *S. mansoni* fucosyltransferases

**Figure 2:**
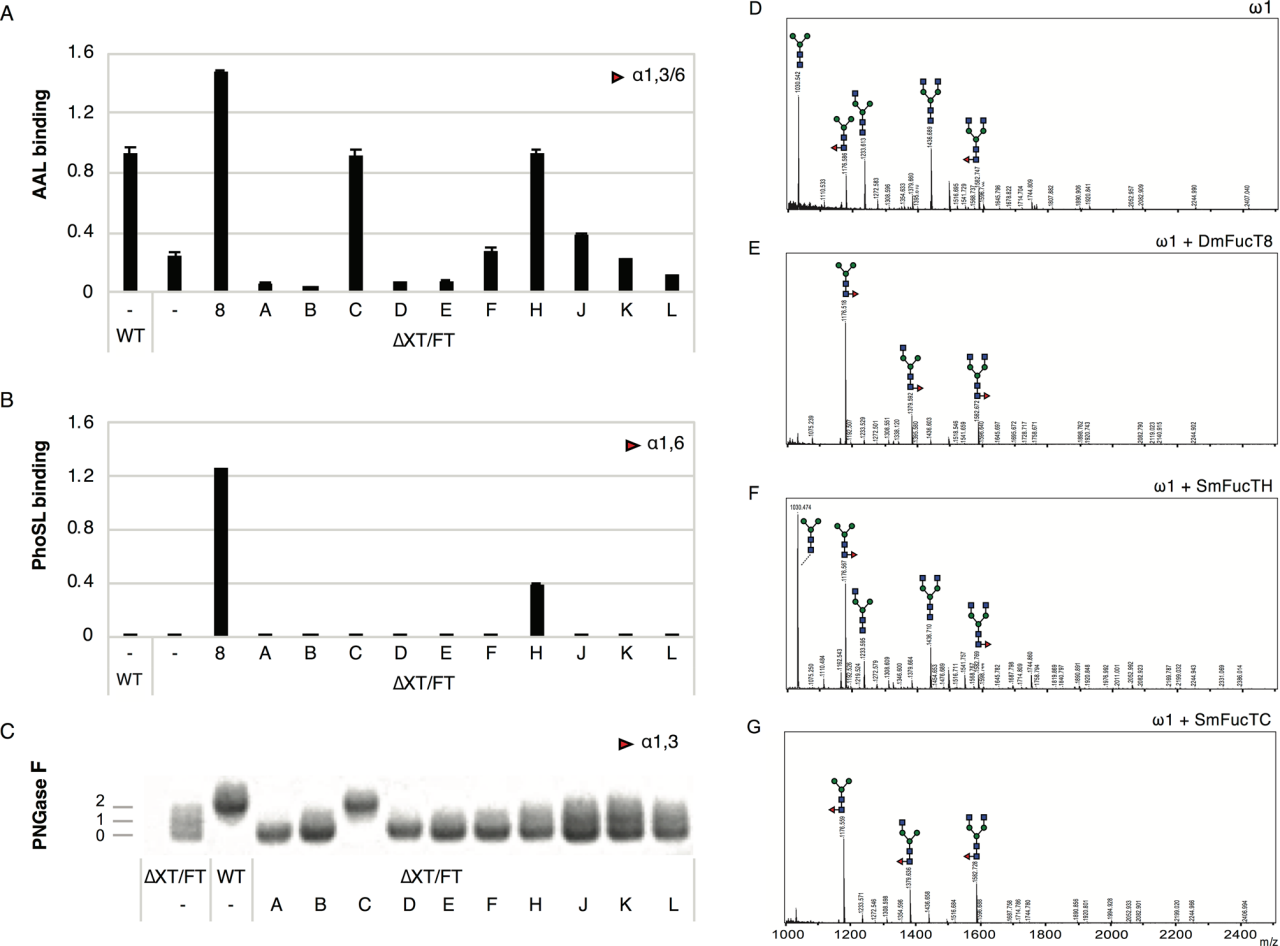
Core fucosylation of omega-1 N-glycans by fucosyltransferases of *S. mansoni* (SmFucTs). Omega-1 was co-expressed in wild-type (WT) or in ΔXT/FT *N. benthamiana* plants with SmFucTs (A to F, H and J to L). After extraction and subsequent purification from the apopolast fluid the glycan composition of omega-1 was analyzed. (**A**) *Aleuria aurantia* lectin (AAL) binding assay for detection of α1,3- and α1,6-linked fucose. (**B**) *Pholiota squarrosa* lectin (PhoSL) binding assay for the specific detection of α1,6-linked fucose. (**C**) Oriole stained SDS-PAGE gel with 200 ng PNGase F treated omega-1 to assess the presence of α1,3-linked core fucose. The number of N-glycans still attached to omega-1 is indicated. (**D**–**G**) MALDI-TOF MS of N-glycans released from omega-1 expressed in ΔXT/FT plants (**D**), or upon co-expression with SmFucTC (**E**), DmFucT8 (**F**) or SmFucTH (**G**).

To investigate which of the SmFucTs can add a fucose to the N-glycan core, each of the SmFucTs or the core α1,6-FucT from *Drosophila melanogaster* (DmFucT8) were co-expressed with omega-1 as carrier glycoprotein in ΔXT/FT *N. benthamiana* plants, which are deficient for N-glycan β1,2-xylosylation and core α1,3-fucosylation. Additionally, omega-1 was expressed in wild-type plants as a control for core α1,3-fucosylation. Omega-1 was purified from the leaf apoplast and fucosylation of omega-1 N-glycans was analyzed with the fucose binding lectin AAL. As expected, only weak binding was observed for the N-glycans of omega-1 produced in ΔXT/FT plants and was therefore considered background (Fig. 2A). On the contrary, strong binding was observed to the N-glycans of omega-1 produced in wild-type or ΔXT/FT plants upon co-expression of DmFucT8. Upon co-expression of SmFucTs, clear AAL binding to the N-glycans of omega-1 was only observed upon co-expression of SmFucTC and SmFucTH, which suggests that these enzymes add a core fucose to the N-glycans of omega-1.

To distinguish whether SmFucTC and SmFucTH add an α1,3- or α1,6-linked fucose to the N-glycan core, another binding assay was performed with the PhoSL lectin, which specifically binds to core α1,6-fucose. This assay revealed that SmFucTH is able to transfer an α1,6-linked fucose to the core of omega-1 N-glycans, although less efficient than the positive control DmFucT8 (Fig. 2B).

Furthermore, mass spectrometry (MS) analysis of PNGase A released N-glycans of omega-1 co-expressed with DmFucT8 or SmFucTH confirmed the addition of fucose (Fig. 2D–F). The major N-glycan found on omega-1 produced in ΔXT/FT plants is the paucimannosidic glycan visible at m/z 1030 Dalton (Fig. 2D), whereas both DmFucT8 and SmFucTH produce a fucosylated paucimannosidic N-glycan detected at m/z 1176 Dalton as the major structure on omega-1 (Fig. 2E,F). MS analysis of omega-1 N-glycans upon co-expression of SmFucTF, SmFucTJ, SmFucTK or SmFucTL showed that these FucTs do not add a core fucose (Fig. S3).

To confirm that SmFucTC adds an α1,3-linked fucose to the N-glycan core, purified omega-1 samples were treated with PNGase F and visualized on an Oriole-stained SDS-PAGE gel. PNGase F releases N-glycans only in the absence of core α1,3-fucose. The release of N-glycans from omega-1 results in a shift in protein size (from 28 to 25 kDa), which is clearly visible when comparing omega-1 samples from wild-type and ΔXT/FT plants (Fig. 2C). Upon co-expression of SmFucTs, N-glycans on omega-1 were only protected against PNGase F cleavage upon co-expression of SmFucTC. This confirms that SmFucTC adds an α1,3-linked fucose to the core of omega-1 N-glycans. MS analysis confirmed the addition of fucose to the majority of omega-1 N-glycans (Fig. 2G), which is comparable to the degree of fucosylation on omega-1 N-glycans produced in wild-type plants^19^.

Altogether, we conclude that SmFucTC and SmFucTH are responsible for the introduction of an α1,3- or α1,6-linked fucose to the core of N-glycans, respectively.

### Lewis X synthesis by *S. mansoni* fucosyltransferases

**Figure 3:**
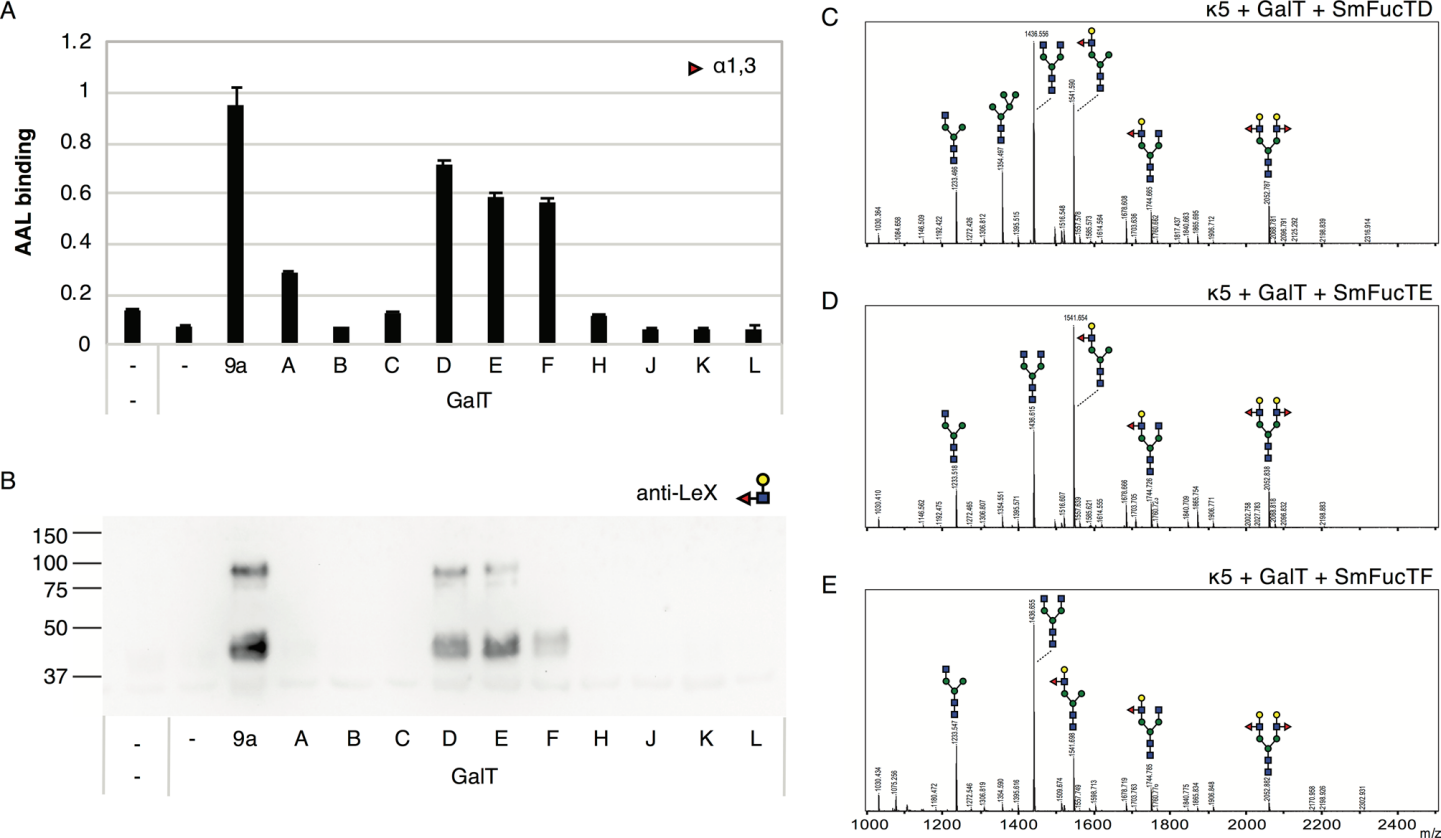
Synthesis of LeX on kappa-5 N-glycans by fucosyltransferases of *S. mansoni* (SmFucTs). Kappa-5 was co-expressed in ΔXT/FT *N. benthamiana* plants with sialDrGalT (GalT) and SmFucTs (A to F, H and J to L) or sialTnFucT9a as a positive control. After extraction of apoplast proteins the glycan composition was analyzed. (**A**) *Aleuria aurantia* lectin (AAL) binding assay for detection of α1,3-linked fucose. (**B**) LeX western blot to reveal the presence of LeX. (**C**–**E**) MALDI-TOF MS profiles of N-glycans released from purified kappa-5 upon co-expression of sialDrGalT with SmFucTD (**C**), SmFucTE (**D**) or SmFucTF (**E**).

To identify which of the SmFucTs is able to synthesize LeX, we co-expressed the carrier glycoprotein kappa-5 with a chimeric galactosyltransferase (sialDrGalT) in ΔXT/FT *N. benthamiana* plants in order to synthesize β1,4-galactose extended branches. Each SmFucT or a chimeric fucosyltransferase (sialTnFucT9a; positive control) was then included to check for LeX synthesis. Kappa-5 was extracted from the leaf apoplast and screened for fucosylated glycan motifs with the lectin AAL. As expected, AAL binds to kappa-5 N-glycans when LeX is synthesized upon co-expression of sialDrGalT and sialTnFucT9a (Fig. 3A). Upon co-expression of SmFucTs strong binding of AAL to the N-glycans of kappa-5 was found for SmFucTD, SmFucTE and SmFucTF, and to a lower extent for SmFucTA.

To check LeX synthesis, a western blot was performed on kappa-5 apoplast samples using an anti-LeX antibody. The western blot revealed two clear bands (at 45 and 90 kDa for monomeric and dimeric kappa-5, respectively) upon co-expression of sialDrGalT with sialTnFucT9a, SmFucTD or SmFucTE (Fig. 3B). A fainter signal for monomeric kappa-5 was observed upon co-expression of SmFucTF, which indicates that this enzyme is able to synthesize LeX but is less efficient than SmFucTD and SmFucTE. No signal for LeX was found upon co-expression of SmFucTA. To confirm synthesis of LeX MS analysis of N-glycans released from purified kappa-5 was performed. Enzymatic treatments with α1,3/4-fucosidase and β-galactosidases (either specific for β1,4/6- or β1,3-linked galactose) were included to confirm the presence of LeX (Figs. S4 and S5). Co-expression of sialDrGalT and sialTnFucT9a allows synthesis of single-branched LeX N-glycans on omega-1 as previously reported^19^. Similarly, significant peaks for single-branched LeX N-glycans were detected upon co-expression of SmFucTD and SmFucTE (detected at 1541 Dalton; Fig. 3C,D). Co-expression of SmFucTF only resulted in a minor peak for a single-branched LeX glycan (Fig. 3E), confirming the weaker signal in the western blot. A similar N-glycan MALDI-TOF MS profile was obtained upon co-expression of SmFucTA (Fig. S6), but in combination with the reduced AAL binding and lack of western blot signal these data are inconclusive regarding the absence or presence of LeX.

Altogether, we conclude that LeX can be synthesized by SmFucTD, SmFucTE, and to lesser extent SmFucTF.

### Fucosylation of LDN by *S. mansoni* fucosyltransferases

**Figure 4:**
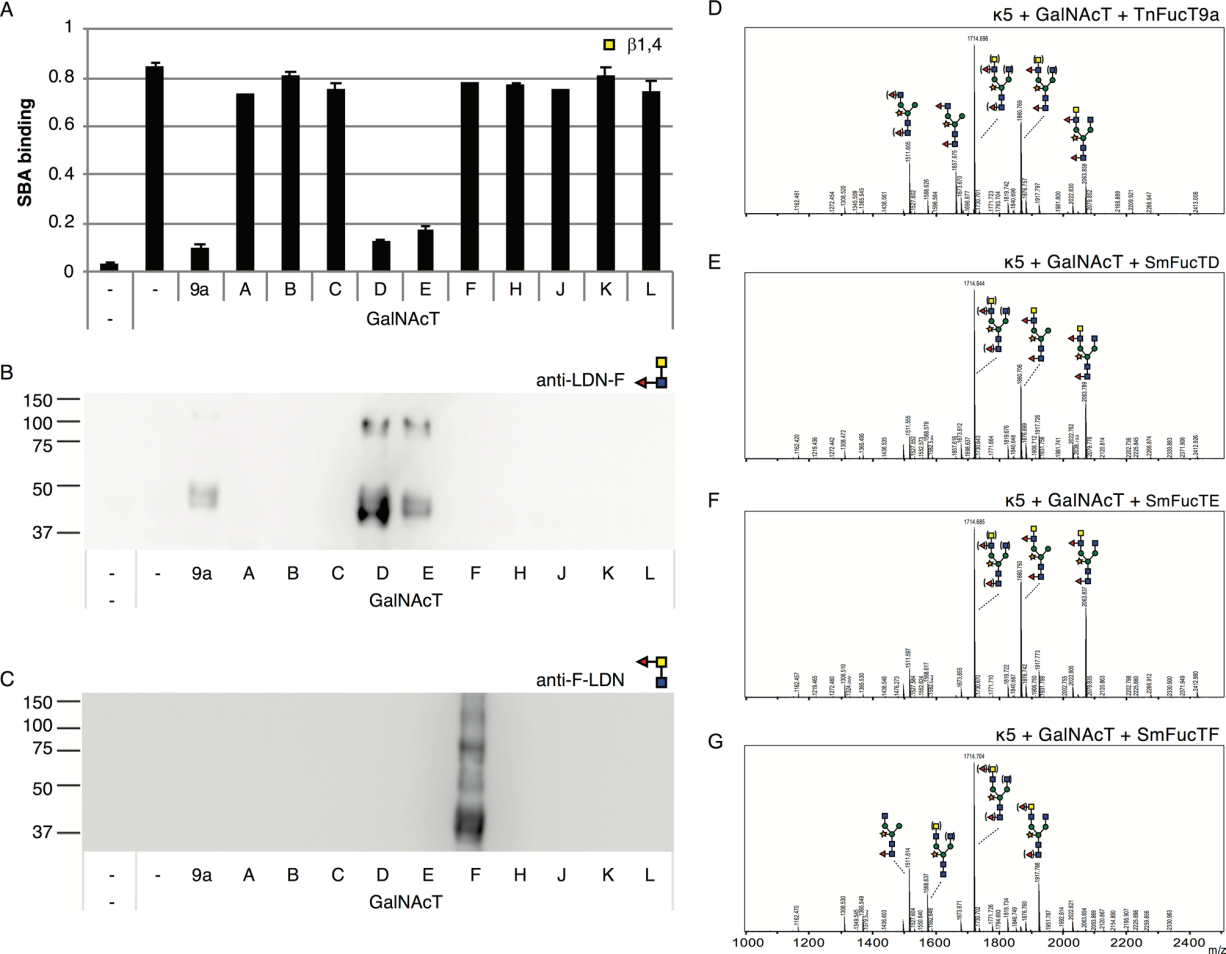
Fucosylation of LDN on kappa-5 N-glycans by fucosyltransferases of *S. mansoni* (SmFucTs). Kappa-5 was co-expressed in wild-type *N. benthamiana* plants with CeGalNAcT and SmFucTs (A to F, H and J to L) or sialTnFucT9a as positive control. After extraction of apoplast proteins the glycan composition was analyzed. (**A**) Soybean agglutinin (SBA) lectin binding assay for detection of GalNAc. (**B**) LDN-F western blot to reveal the presence of LDN-F. (**C**) F-LDN western blot to reveal the presence of F-LDN. (**D**–**G**) MALDI-TOF MS profiles of N-glycans released from purified kappa-5 upon co-expression of CeGalNAcT with sialTnFucT9a (**D**), SmFucTD (**E**) SmFucTE (**F**) SmFucTF (**G**). When a MS peak represents multiple N-glycan structures of identical mass, the possible positions of these sugar residues on the N-glycan are indicated between brackets.

To identify which of the SmFucTs are able to fucosylate LDN (antennary GalNAcβ1-4GlcNAc), kappa-5 was co-expressed with CeGalNAcT in wild-type *N. benthamiana* plants in order to synthesize β1,4-GalNAc extended N-glycan branches. SmFucTs or the positive control sialTnFucT9a were then included to check for the introduction of a fucose on LDN. Kappa-5 was extracted from the leaf apoplast and screened for fucosylated LDN motifs with the SBA lectin, which will show reduced binding to LDN upon fucosylation. As expected, SBA bound strongly to the N-glycans of kappa-5 upon co-expression of CeGalNAcT, whereas the combination with sialTnFucT9a completely abolished binding of SBA (Fig. 4A). Similarly, SBA binding to kappa-5 N-glycans was strongly inhibited upon co-expression of SmFucTD and SmFucTE.

To check whether SmFucTD and SmFucTE synthesize LDN-F or F-LDN (Fucα1-3GalNAcβ1-4-GlcNAc), first a western blot was performed with an anti-LDN-F antibody (Fig. 4B). The western blot revealed a faint signal for kappa-5 upon co-expression of CeGalNAcT with the positive control sialTnFucT9a. Clear bands for kappa-5 were detected upon co-expression of SmFucTD or SmFucTE, indicating that both enzymes are able to synthesize LDN-F. Next, we performed a western blot with an anti-F-LDN antibody in order to identify SmFucTs that synthesize F-LDN (Fig. 4C). A clear signal was observed for F-LDN upon co-expression of SmFucTF. This was surprising, since co-expression of SmFucTF did not block SBA binding, which therefore suggests partial conversion to F-LDN.

MS analysis was then performed on released N-glycans of purified kappa-5 to confirm synthesis of either LDN-F or F-LDN. Enzymatic treatments with β-*N*-acetyl-glucosaminidase and β-*N*-acetyl-hexosaminidase were included to confirm the presence of fucosylated LDN (Fig. S7). As expected, co-expression of CeGalNAcT and sialTnFucT9a allowed synthesis of N-glycans with a single LDN-F motif (detected at 1714 Dalton; Figs. 4D and S4A) as previously reported^19^. Additionally, we detected a significant proportion of N-glycan structures with a fucosylated terminal GlcNAc residue (detected at 1657 Dalton; Fig. 4D, Fig. S7A). Upon co-expression of SmFucTD and SmFucTE, N-glycans with a single LDN-F motif were also detected (Fig. 4E,F, respectively). Interestingly, combined glucosaminidase and hexosaminidase treatment revealed that in contrast to the positive control sialTnFucT9a, the majority of N-glycans synthesized by SmFucTD and SmFucTE carried LDN-F. Furthermore, we did not detect N-glycan structures with a fucosylated terminal GlcNAc residue (Fig. S7B,C). This indicates that SmFucTD and SmFucTE are more efficient in synthesizing LDN-F compared to sialTnFucT9a. Lastly, MS analysis suggested that only a small portion of N-glycans can be synthesized with a single F-LDN motif upon co-expression of SmFucTF (Fig. 4G), but this glycan motif was completely lost after combined glucosaminidase and hexosaminidase treatment (Fig. S7D). This suggests that only a minor fraction of F-LDN is synthesized by co-expression of SmFucTF.

Altogether, we conclude that LDN-F can be synthesized by SmFucTD and SmFucTE, whereas SmFucTF could be involved in the synthesis of F-LDN.

### Synthesis of F-LDN-F by *S. mansoni* fucosyltransferases

**Figure 5:**
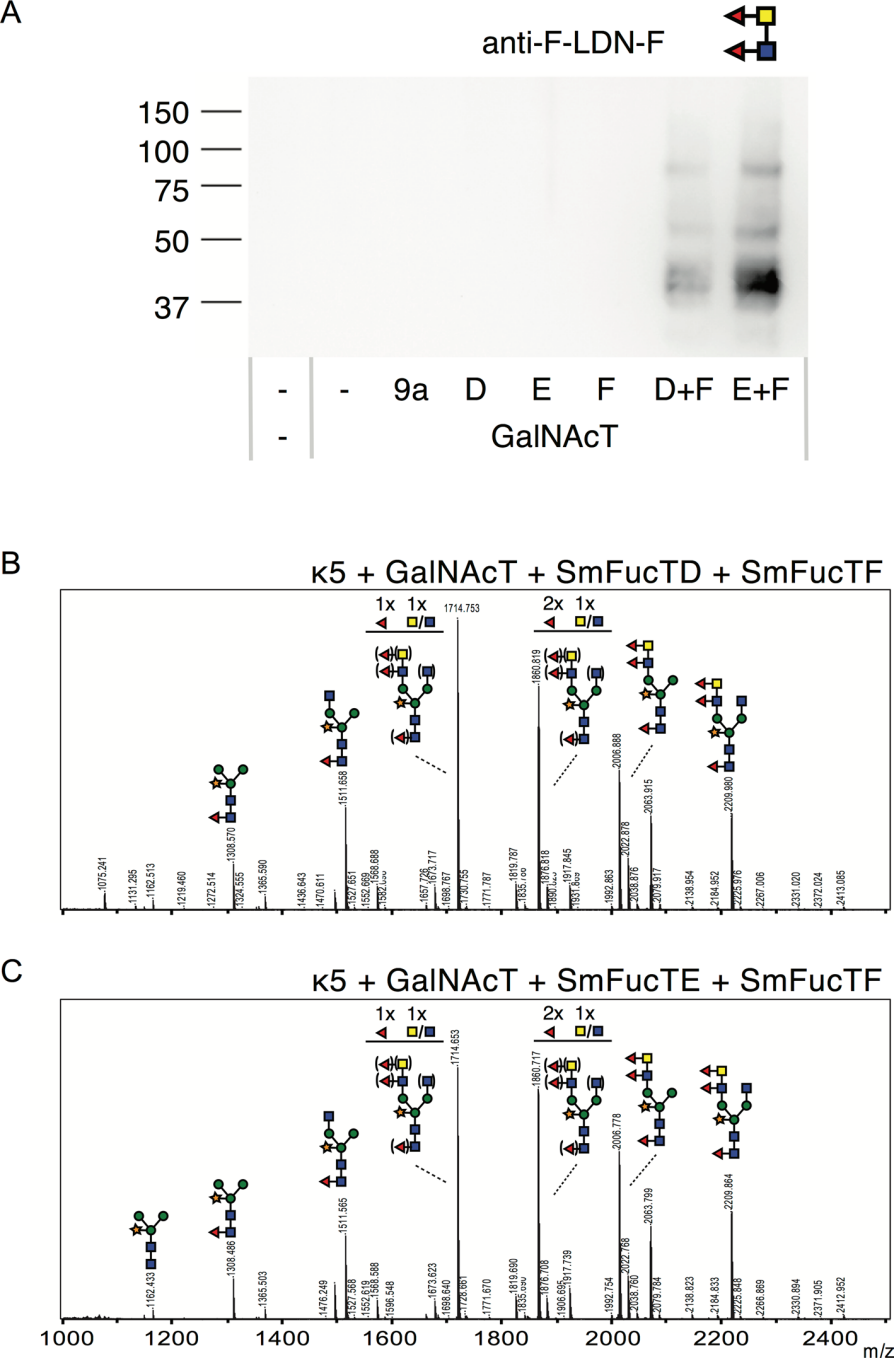
Synthesis of F-LDN-F on kappa-5 N-glycans by fucosyltransferases of *S. mansoni* (SmFucTs). Kappa-5 was co-expressed in wild-type *N. benthamiana* plants with CeGalNAcT and combinations of SmFucTD, SmFucTE and SmFucTF. Co-expression of sialTnFucT9a was included as a negative control. After extraction and subsequent purification from the apoplast fluid the glycan composition of kappa-5 was analyzed. (**A**) F-LDN-F western blot to reveal the presence of F-LDN-F. (**B**,**C**) MALDI-TOF MS profiles for N-glycans of kappa-5 upon co-expression of SmFucTF with SmFucTD (**B**) or SmFucTE (**C**). When MS peaks represent multiple N-glycan structures of identical mass, the number of monosaccharide residues for which the position on the N-glycan is not clear is indicated above the glycan structure and the possible variations are indicated between brackets.

The identification of the SmFucTs responsible for LDN-F synthesis and the indication of F-LDN synthesis by SmFucTF allowed us to examine whether the F-LDN-F motif could be synthesized on the N-glycans of kappa-5. Therefore, we co-expressed kappa-5 in wild-type *N. benthamiana* plants with CeGalNAcT and sialTnFucT9a, SmFucTD, SmFucTE or SmFucTF, or a combination of SmFucTF with SmFucTD or SmFucTE. After purification of kappa-5, the glycan composition was analyzed by a western blot with an anti-F-LDN-F antibody (Fig. 5A). This blot revealed multiple protein bands around 45, 55 and 90 kDa upon co-expression of SmFucTF with SmFucTD or SmFucTE. To confirm the synthesis of F-LDN-F, MS analysis was performed and significant proportions of N-glycans with F-LDN-F were detected for both samples (Fig. 5B,C). Furthermore, combined glucosaminidase and hexosaminidase treatment confirmed that the majority of the N-glycans on kappa-5 carried either LDN-F or F-LDN-F (detected at 1860 and 2007 Dalton, respectively; Fig. S8). These results reveal that SmFucTF is involved in the synthesis of the F-LDN-F motif together with SmFucTD or SmFucTE.

## Discussion

**Figure 6:**
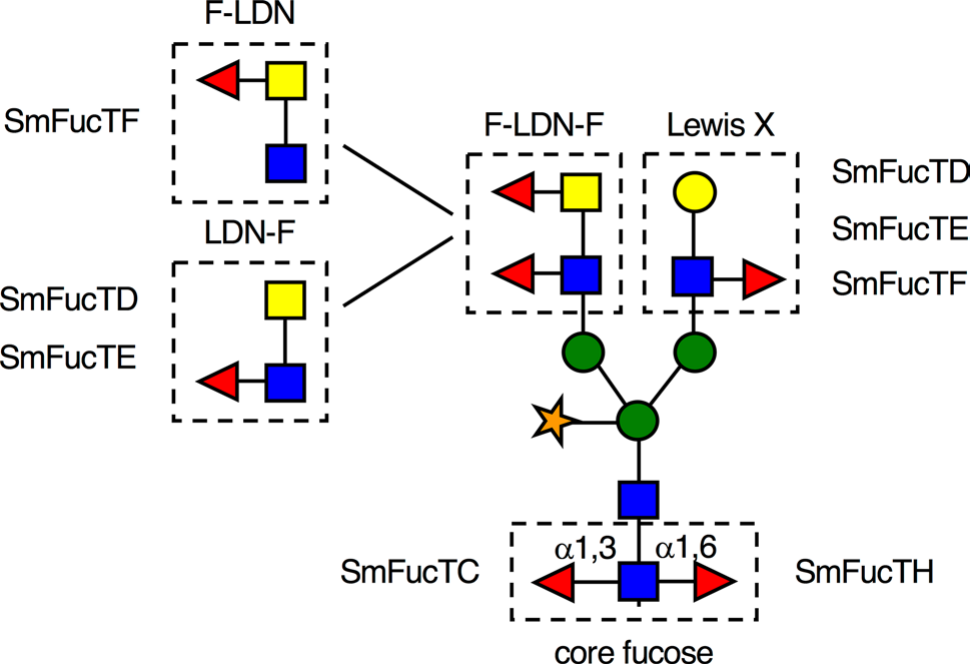
Graphical representation of glycan motifs synthesized by *S. mansoni* fucosyltransferases (SmFucTs).

After sequencing of the *S. mansoni* genome in 2009 various putative SmFucT coding sequences were found, of which 14 full-length genes could be amplified from cDNA^20,25^. Currently, these SmFucTs are characterized based on sequence analysis^20^ and SmFucTF is the only functionally characterized enzyme based on chemoenzymatic assays^21^*.* However, characterization based on sequences or chemoenzymatic assays can differ from the in vivo biological function. Therefore, ten selected SmFucTs were characterized using a plant-based system, to understand how certain fucosylated N-glycan motifs are synthesized in vivo. Since, sub-Golgi localization of a FucT can be an important indicator for its function, we first determined Golgi distribution of the ten SmFucTs. This study revealed subtle differences in sub-Golgi localization of the selected SmFucTs. Thereafter, the SmFucTs were co-expressed with carrier glycoproteins in *N. benthamiana* and the N-glycan composition of the carrier glycoproteins was analyzed for fucosylated glycan motifs. With this new approach we were able to functionally characterize five out of ten SmFucT enzymes (Fig. 6). Furthermore, we attribute a new function to SmFucTF, since it is involved in the synthesis of F-LDN-F besides LeX^21^.

Fucosylated glycan motifs are differentially expressed during the lifecycle of *S. mansoni* and are most abundant in the egg stages of the parasite^2,4^. Several studies have analyzed the transcription levels of multiple SmFucTs in order to predict which enzyme is responsible for the synthesis of specific glycan motifs. However, comparison between these studies is difficult because of selected life stages and recurrent changes in accession numbers^20,21,26^. An extensive transcriptomic life cycle analysis performed by Fitzpatrick and colleagues showed differential expression of smp_054300, smp_137740 and smp_137730^26^, which are sequences related to SmFucTD, SmFucTE and SmFucTF, respectively. SmFucTD is scarcely expressed in cercariae, schistosomula and young worms, whereas SmFucTE and SmFucTF are clearly expressed in all life stages. More recently, a web portal for gene expression across all life stages of *Schistosoma mansoni* has been made available (Table S2)^27^. From this meta-analysis it can be deduced that SmFucTE (smp_133740) and SmFucTF (now smp_142860) are both highly expressed in the egg stage of *S. mansoni*, which coincides with the high abundance of fucosylated glycan motifs in parasite eggs^4^. SmFucTD on the other hand is specifically expressed by the miracidium and sporocysts, which suggest that it plays a role in the synthesis of LDN-F, a glycan motif commonly found in these parasite life stages^2,3^. In addition to enzymes that presumably modify the glycan antenna, expression profiles can be retrieved for the SmFucTs that modify the N-glycan core. Both SmFucTC (smp_154410) and SmFucTH (smp_175120) are expressed throughout the *S. mansoni* life cycle, where the core α1,3-SmFucTC is most abundant in cercariae and schistosomula, followed by the eggs. The expression of the core α1,6-SmFucTH on the other hand is abundant in cercariae and increases upon development into schistosomula and adults. This indicates that the presence of core α1,6-fucose coincides with the life stages in the human host.

In the glycosylation pathway of *S. mansoni* and other invertebrates fucosylation appears to occur via strictly ordered FucTs^28^. Sub-Golgi localization analysis of SmFucTs revealed subtle differences in the degree of co-localization with three Golgi markers. For instance, the α1,6-SmFucTs showed a high Pearson’s correlation coefficient with each of the three markers and no significant difference in co-localization, suggesting localization through the entire Golgi. SmFucTH and SmFucTJ to L seem to localize more with the *cis*/medial-Golgi than the α1,3-SmFucTs. Therefore, these enzymes are likely α1,6-SmFucTs, since α1,6-fucosylation is expected to occur prior to α1,3-fucosylation. In comparison to the other α1,3-SmFucTs, SmFucTC localized more with the medial-Golgi, which could suggest a role in core α1,3-fucosylation. This is in agreement with medial/*trans*-Golgi localization observed for the plant core α1,3-FucT and the functional prediction for SmFucTC made by Mickum and colleagues^21,29–31^. The remaining SmFucTs (A, B, D, E and F) localized more to the *trans*-Golgi and are therefore likely involved in the synthesis of more complex glycan motifs. These α1,3-SmFucTs were therefore expected to be involved in the synthesis of more complex glycan motifs such as LDN-F, LeX and DF, which is in agreement with the predictions of Mickum and colleagues^21^.

After Golgi localization was confirmed, we investigated core fucosylation on carrier glycoprotein omega-1 and identified SmFucTC as a core α1,3-FucT, which agreed with our prediction based on its localization. Additionally, SmFucTH was identified as core α1,6-FucT. Since core fucosylation is the only known motif with an α1,6-linked fucose, the function of the other three putative α1,6-SmFucTs SmFucTJ, SmFucTK and SmFucTL remains unknown. Still, it is striking that four of the ten selected SmFucTs are assigned as α1,6-SmFucTs based on sequence analysis^20^. Peterson and colleagues suggested that a secondary function could explain the large number of α1,6-SmFucTs^20^. Furthermore, depending on the protein environment, the α1,6-SmFucTs may differ in their capacity to add an α1,6-fucose. So, SmFucTJ, SmFucTK and SmFucTL may be involved in the core α1,6-fucosylation of glycoproteins other than omega-1 or glycoproteins in different life stages. Although protein environment is thought to influence specific glycan modifications, site-specific activity for glycosyltransferases with the same function has never been shown^32–34^.

LeX is one of the fucosylated motifs found on antennae of *S. mansoni* N-glycans, which can be synthesized in *N. benthamiana* by co-expression of sialDrGalT and sialTnFucT9a^19^. For *S. mansoni*, Mickum and colleagues showed that SmFucTF can synthesize LeX on different glycan acceptors^21^. In this study we show that SmFucTF indeed is involved in the synthesis of LeX in vivo. However, besides SmFucTF, we also found that SmFucTD and SmFucTE are able to synthesize LeX on N-glycans. Sequence comparison of these three SmFucTs reveals that SmFucTF only shares 41.6% and 69.6% sequences similarity with SmFucTD and SmFucTE, respectively. This could suggest that although SmFucTD, SmFucTE and SmFucTF create the same glycosidic linkage, they may have different acceptor specificities for N-glycans, O-glycans and glycolipids. In the study of Mickum and colleagues, SmFucTF also showed specificity to different glycan acceptors^21^. Moreover, SmFucTD, SmFucTE and SmFucTF may be active in different life stages or tissues. So, differences in life stage expression combined with glycan acceptor specificities can explain why *S. mansoni* has three SmFucTs for the synthesis of LeX.

Other complex motifs found on N-glycans of *S. mansoni* are fucosylated LDN structures, like LDN-F and F-LDN. LDN-F was previously synthesized in *N. benthamiana* by co-expression of CeGalNAcT and sialTnFucT9a^19^. In the current study we show that SmFucTD and SmFucTE are able to synthesize LDN-F (besides LeX), whereas SmFucTF is able to synthesize F-LDN. N-glycan profiles show that F-LDN synthesis by SmFucTF is very inefficient, but co-expression of SmFucTD or SmFucTE enables the synthesis of a significant portion of F-LDN-F on N-glycans of the carrier protein kappa-5. Moreover, a detailed glycomics analysis of *S. mansoni* performed by Smit and colleagues showed that fucosylation of GalNAc in the LDN motif is mostly occurring in combination with GlcNAc fucosylation, forming F-LDN-F^3^. This suggests that SmFucTF prefers LDN-F as glycan acceptor over LDN. Restricted acceptor use may also explain Mickum and colleagues were unable to show fucosylation of LDN by SmFucTF in their experiments with the glycan acceptor GalNAcβ1–4GlcNAcβ1–3Galβ1–4Glc-AEAB^21^. This glycan acceptor is not a natural glycan acceptor, whereas the engineered N-glycan with a single LDN antennae in plants is also present in *S. mansoni*^3^. Furthermore, the presence of SmFucTD or SmFucTE could be required for proper fucosylation by SmFucTF of N-glycan associated LDN.

In *S. mansoni* the LDN-F and F-LDN-F motifs can be substituted with additional fucoses to form the LDN-DF (GalNAcβ1–4(Fucα1–2Fucα1–3)GlcNAc) and DF-LDN-DF (Fucα1–2Fucα1–3GalNAcβ1–4(Fucα1–2Fucα1–3)GlcNAc) motifs. This DF motif is synthesized by an α1,2-SmFucT, which links two fucoses via an α1,2-bond. None of the SmFucTs was initially characterized as α1,2-SmFucT, therefore the α1,2-SmFucT could be among the putative α1,3- or α1,6-SmFucTs. Unfortunately, co-expression of the selected SmFucTs with different combinations of glycosyltransferases, hexosaminidases and glycoproteins (to generate different glycan acceptor structures) did not result in detectable DF formation (data not shown). Furthermore, the DF motif is not commonly found on N-glycans in *S. mansoni* but is more abundant on O-glycans and glycolipids^3,6^. Hence, functional characterization of SmFucTs in plants with O-glycan acceptor structures would be a logical next step.

Unfortunately, we were not able to reveal conclusive evidence for SmFucTA, B, J, K and L activity in any of our experiments. We did observe some activity for SmFucTA in our Lewis X screening, where a minor fraction of kappa-5 N-glycans could carry the LeX motif. However, our AAL binding assay and LeX western blot did not support this conclusion. This could suggest that N-glycans are not the most optimal glycan acceptor for LeX synthesis by SmFucTA, whereas glycolipids or O-glycans could be more suitable. An alternative would be that SmFucTA substitutes β1,4-galactose with fucose and in this way is involved in the synthesis of the glycan motif pseudo-LeY (Fucα1-3Galβ1-4(Fucα1-3)GlcNAc), which is unique to glycolipids of cercariae and early schistosomula^3^. Based on the RNAseq meta-analysis, the expression level of SmFucTA (smp_214370) is upregulated in cercariae and continues to increase until early adults (Table S2). This could support a role for pseudo-LeY synthesis. Future work should now address the activity of SmFucTA towards glycolipid substrates and whether it is involved in LeX or pseudo LeY synthesis.

The remaining SmFucTs (B, J, K and L) could then be involved in formation of the DF glycan motif. The α1,6-FucT sequence motifs I and II, as described by Breton and colleagues^35^, are shared between the α1,6-FucTs and the known α1,2-FucTs adding an α1,2-fucose to a galactose residue^36^. This suggests that one of the remaining α1,6 SmFucTs could be involved in DF synthesis. On the other hand, sequence comparison by Mickum and colleagues^21^ suggests that SmFucTB (and SmFucTA) could be involved in synthesis of the DF motif as well. Another option could be that none of the selected SmFucTs is able to synthesize DF and one of the many splice variants found among the SmFucTs encodes the missing α1,2-SmFucT^20^.

In this study we revealed the biological function of five SmFucTs. This knowledge can be used to unravel the role of fucosylated glycans in parasite biology and host–parasite interactions. With further progress being made in *S. mansoni* gene manipulation methodology it will be interesting to study the effects of prevention of expression, or overexpression of these enzymes. For example, targeted disruption of the expression of functional enzymes involved in the synthesis of LeX and LDN-F would allow investigation on their role in host–parasite interaction. Additionally, we established a new in vivo platform for the functional characterization of glycosyltransferases using *N. benthamiana* plants. The next step would be to further characterize SmFucT activity using O-glycans or glycolipids as acceptor substrates. Furthermore, the functionally characterized SmFucTs can directly be applied to synthesize glycoproteins with native fucosylated N-glycans in order to study their role in parasite biology or as immunotherapeutic molecules as was done previously to illustrate the importance of LeX on the Th2 polarizing properties of omega-1^19^. Ultimately, these glyco-engineered glycoproteins can be used in immunological studies to investigate application as vaccines or as biopharmaceuticals.

## Material and methods

### Selection of *Schistosoma mansoni* fucosyltransferases

For the functional characterization of *S. mansoni* fucosyltransferases (SmFucTs) we found up to 50 putative genes in literature or databases, like Uniprot and GeneDB (Table S1). Until now only 15 of these putative genes were amplified from *S. mansoni* cDNA^20^. Two of these FucTs (POFucTA and B) were classified as enzymes involved in protein O-fucosylation and therefore excluded for functional characterization on N-glycans. The other enzymes (SmFucTA to M) were predicted to be involved in α1,3- or α1,6-fucosylation. SmFucTG was excluded as it encodes a premature stop codon and lacks a transmembrane region^20^, which is characteristic for type II transmembrane Golgi proteins and proper Golgi localization. The similarity on amino acid level between the α1,6-SmFucTs (H to M) was remarkably higher compared to the α1,3-SmFucTs (A to F). SmFucTI and SmFucTM were also excluded from further analysis, because the similarity between SmFucTI, J and M was above 89% and the observed mutations were not present in conserved protein regions shared by α2-, α6- and protein O-FucTs^20^.

### Construction of expression vectors

The full-length open reading frames of SmFucTs (A to F, H and J to L) were codon optimized in-house, flanked by a NcoI/BspHI restriction site at the 5′-end and a KpnI restriction site at the 3′-end, and synthetically constructed at GeneArt. For SmFucTA an extra alanine residue was introduced after the start codon to introduce a NcoI restriction site. The SmFucTs were then cloned into the plant expression vector pHYG via the NcoI/BspHI and KpnI restriction sites^37^.

For confocal studies green fluorescent protein (GFP)-tagged SmFucTs were constructed in the pHYG plant expression vector. The SmFucT sequences were reamplified by PCR to introduce a GGGGS-linker and a NheI restriction site at the 3′ end in order to clone the SmFucTs in frame with a C-terminal GFP fragment in pHYG. For sub-Golgi localization we used three red fluorescent protein (RFP)-tagged Golgi reference markers: β1,2-*N*-acetyl-glucosaminyltransferase I (GnTI) from *Nicotiana tabacum* fused to RFP (GnTI-RFP), β1,2-xylosyltransferase from *Arabidopsis thaliana* fused to RFP (XylT-RFP) and the CTS domain of *Rattus norvegicus* α2,6-sialyltransferase fused to the catalytic domain of *N. tabacum* GnTI and RFP (ST-RFP)^38–40^.

To determine SmFucT activity, constructs with the carrier glycoproteins omega-1 or kappa-5 were used^19^. For screening purposes we used a hybrid β1,4-galactosyltransferase from *Danio rerio* (sialDrGalT) under the control of the GPAII promoter in the pHYG expression vector or a β1,4-*N*-actylgalactosaminyltransferase from *Caenorhabditis elegans* (CeGalNAcT) in the pBINPLUS expression vector to synthesize β1,4-galactose or β1,4-galactosamine (GalNAc) extended branches, respectively^19^. As positive controls we used a hybrid α1,3-fucosyltransferase 9a from *Tetraodon nigriviridus* (sialTnFucT9a) to synthesize LeX and LDN-F, and core α1,6-fucosyltransferse from *Drosophila melanogaster* (DmFucT8) both in the pBIN-PLUS vector^19,41^.

All constructs (unless stated otherwise) were driven by the Cauliflower mosaic virus 35S promoter with duplicated enhancer (d35S) and the *Agrobacterium tumefaciens* nopaline synthase transcription terminator (Tnos). Furthermore, to boost translation a 5ʹ leader sequence of the alfalfa mosaic virus RNA 4 (AIMV) was included between the promoter and the construct. The P19 silencing suppressor from tomato bushy stunt virus pBIN61 was used to enhance expression, unless stated otherwise^42^. For plant expression, all constructs were transformed into *A. tumefaciens* strain MOG101 except GnTI-RFP, XylT-RFP and ST-RFP that were transformed into GV3101.

### Agroinfiltration

*Agrobacterium tumefaciens* cultures were grown at 28 °C/250 rpm in LB medium (10 g/L peptone140, 10 g/L NaCl, 5 g/L yeast, pH 7.0), with 50 µg/mL kanamycin and 20 µM acetosyringone. After overnight (o/n) incubation, bacterial cultures were centrifuged for 15 min/2880×*g*. For Golgi localization, bacteria were washed twice in 1 mL infiltration medium (50 mM MES, 2 mM Na_3_PO_4_·12H_2_O, 0.1 mM acetosyringone and 5 mg/ml glucose) and resuspended in infiltration medium. For protein production, bacteria were directly resuspended in MMA (1.95 g/L MES, 20 g/L sucrose, 5 g/L MS-salts, 0.2 mM acetosyringone, pH 5.6). *A. tumefaciens* cultures of different constructs were mixed for co-expression and the final optical density of each *A. tumefaciens* culture in the mixture varied between 0.1 and 0.5, depending on the construct. The youngest fully expanded leaves of 4 to 6 week old *Nicotiana benthamiana* plants (wild-type or ΔXT/FT plants^24^) were infiltrated at the abaxial side. ΔXT/FT plants are generated by the stable integration of a hairpin construct that facilitates stable down-regulation of endogenous plant FucTs by RNA interference. For Golgi localization studies, plants were grown in a controlled greenhouse compartment (Oxford Brooks University) and after infiltration grown in incubators at 21 °C (14 h light and 10 h darkness). For protein production, plants were maintained throughout the experiment in a controlled greenhouse compartment (UNIFARM, Wageningen).

### Golgi localization

Three days post infiltration (dpi) leaf epidermal cells were studied with confocal microscopy. High resolution movies were acquired with the Zeiss time series software on the Zeiss LSM 880 confocal microscope with Airyscan detectors (Zeiss) using a 100 × 1.46NA lens. Images were acquired with 8× digital zoom, dual emission filters 500-550BP and 565LP, chlorophyll autofluorescence blocking filter 620SP and simultaneous GFP-RFP laser excitation at 488 nm and 561 nm, respectively. Airyscan acquired movies were processed to one picture per frame. From these movies with integration time ~ 0.8 s, frames with side-on view Golgi bodies were selected for analysis with imageJ plugin Coloc. Per combination the Pearson’s correlation coefficient was determined of minimal 16 side-on view Golgi bodies. The Pearson correlation coefficient of analyzed Golgi bodies was visualized in R (v 3.5.0; win × 64) using ggplot. To determine which variables were capturing variance in the data, an ANOVA was performed using R. The model used was Yi ~ SmFucT + marker + ε. Where Y is the calculated Pearson correlation coefficient of the analyzed Golgi body i (1, 2, …., 879), which was explained over GFP tagged-SmFucT (A, B, C, D, E, F, H, J, K or L), RFP-tagged Golgi reference marker (either GnTI, XylT or ST), and error term ε. Subsequently, a post-hoc analysis was performed using a Tukey’s HSD test. The significances reported were corrected for multiple testing. For graphical display, fluorescent intensity profiles were generated across side-on view Golgi stacks using the Profile tool in the ZEN Blue software by drawing a line across dual-labelled Golgi stacks.

### Protein isolation and purification

Five to six dpi leaves were harvested and apoplastic proteins were isolated via apoplast wash. Harvested leaves were submerged in extraction buffer (50 mM phosphate buffer, 0.1 M NaCl, 0,1% v/v Tween-20, pH 8.0), a vacuum was applied and after 5 min slowly released to infiltrate the leaves with buffer. The apoplast fluid was extracted from the leaves by centrifugation for 10 min/2000×*g*. The protein concentration of the apoplast fluid was determined by the Pierce Bicinchoninic Acid Protein Assay (BCA, Fisher Scientific).

Prior to purification, extracted apoplast fluids were passed through Sephadex G25 chromatography columns to exchange extraction buffer for binding buffer (10 mM Sørensen’s phosphate buffer, 0.1 M NaCl, pH 6.0) and subsequently clarified by centrifugation for 5 min/16,000×*g*. Proteins were then bound to Pierce Strong Cation Exchange Mini Spin Columns (Fisher Scientific). Omega-1 was eluted with a 50 mM Tris–HCl buffer containing 2 M NaCl (pH 9.0), whereas kappa-5 was eluted with binding buffer containing 2 M NaCl. Column loading, washing and elution was done by centrifugation for 5 min/2000×*g*. After elution samples were dialyzed against PBS. The protein concentration of the purified proteins was determined by a BCA assay (Fisher Scientific).

### Lectin binding assays

*Aleuria aurantia* lectin (AAL, Bio-Connect) was used to screen for fucosylation in general, whereas *Pholiota squarrosa* lectin (PhoSL) was used to screen specifically for core α1,6-fucose^43,44^. Soybean agglutinin (SBA, Bio-Connect) was used to determine fucosylation of LDN, since fucosylation of LDN is expected to inhibit binding of SBA to the GalNAc residue of LDN. Microtiter plates were coated o/n with apoplast fluids or purified proteins in PBS at a protein concentration of 1 µg/mL for binding by AAL, or 10 µg/mL for binding by PhoSL or SBA. Plates were blocked with carbohydrate-free blocking buffer (Vector Laboratories) for 1 h at room temperature (RT). Plates were then incubated for 1 h at RT with biotinylated lectin at a concentration of 2 µg/mL AAL, 1.8 µg/mL PhoSL or 5 µg/mL SBA. Subsequently, plates were incubated with avidin-HRP (eBioscience) for 30 min at RT. After every incubation step the microtiter plates were washed 5 times with PBST (PBS containing 0.05% v/v Tween-20). Lectin binding was visualized by adding TMB substrate (Fisher Scientific) and absorbance was measured at a wavelength of 450 nm while using 655 nm as reference filter.

### SDS-PAGE and western blots

For screening of core α1,3-fucosylation, purified omega-1 samples were treated with Peptide:N-glycosidase F (PNGase F, New England Biolabs) according to manufacturer’s protocol. Deglycosylation by PNGase F was then analyzed on a 12% Bis–Tris SDS-PAGE gel (Fisher Scientific) followed by Oriole staining (BioRad). For glycan detection by western blot, apoplast fluids or purified kappa-5 samples were run on a 12% Bis–Tris gel under reducing conditions and subsequently transferred to a PVDF membrane by wet blotting. After blotting, the membrane was blocked for 1 h at RT or o/n at 4 °C with 5% w/v bovine serum albumin in PBST (containing 0.1% v/v Tween-20). Next, the membrane was incubated for 1 h at RT or o/n at 4 °C with primary antibodies targeting LDN-F (mouse IgM, clone 290-2E6^45^, F-LDN (mouse IgM, clone 291-5D5^46^), F-LDN-F (mouse IgM , clone 128-1E7^46^), or LeX (rat IgM, clone 5750^47^). Subsequently, the membrane was incubated for 1 h at RT with 0.75 µg/mL HRP-labelled donkey anti-mouse IgM (Jackson ImmunoResearch laboratories). After every incubation step the membrane was washed 5 times with PBST. The anti-HRP labeled antibodies were detected with a 1:1 SuperSignal West Femto:Dura substrate (Fisher Scientific) in the G:BOX Chemi System (Syngene).

### Matrix-assisted laser desorption/ionization time-of-flight mass spectrometry

Glycans of purified omega-1 or kappa-5 were released, purified and labeled with anthranilic acid as previously described^19^ and MALDI-TOF mass spectra were obtained using an Ultraflex II mass spectrometer (Bruker Daltonics) in negative-ion reflection mode. To confirm the presence of specific glycan motifs, N-glycans were treated with enzymes prior to ZipTip C18 clean-up and subsequent MALDI-TOF MS analysis. N-glycans were treated with α(1-3,4)-fucosidase from *Xanthomonas* sp. (Sigma-Aldrich) with Jack bean β(1-4,6)-galactosidase (Prozyme) or *Xanthomonas manihotis* β(1-3,6)-galactosidase (Prozyme) according to the suppliers protocols to confirm the presence of LeX. To confirm the presence of LDN-F, F-LDN or F-LDN-F, N-glycans were treated with β-*N*-acetyl-hexosaminidase from *Streptomyces plicatus* (New England Biolabs) and β-*N*-acetyl-glucosaminidase from *Streptococcus pneumoniae* (New England Biolabs) according to the suppliers’ protocols.

## Supplementary information

Supplementary Figure Legends.

Supplementary Information.
