# Publisher Correction: Functional characterization of *Schistosoma mansoni* fucosyltransferases in *Nicotiana benthamiana* plants

**DOI:** 10.1038/s41598-021-83766-0

**Published:** 2021-02-17

**Authors:** Kim van Noort, Dieu‑Linh Nguyen, Verena Kriechbaumer, Chris Hawes, Cornelis H. Hokke, Arjen Schots, Ruud H. P. Wilbers

**Affiliations:** 1 Laboratory of Nematology, Plant Sciences Group, Wageningen University and Research, Droevendaalsesteeg 1, 6708 PB Wageningen, The Netherlands; 2 Department of Parasitology, Leiden University Medical Center, Albinusdreef, 2333 ZA Leiden, The Netherlands; 3 Department of Biological and Medical Sciences, Oxford Brookes University, Oxford, OX3 0BP UK

Correction to: *Scientific Reports* 10.1038/s41598-020-74485-z, published online 28 October 2020

The Supplementary Information file published with this Article is incorrect, where Tables S2 and Figures S1–S8 were omitted.

The correct Supplementary Information file is linked to this correction notice.

## Supplementary information

Supplementary Information.

