# Supplementary material for: Functional characterization of Schistosoma mansoni fucosyltransferases in Nicotiana benthamiana plants
Source: Sci Rep. 2020 Oct 28;10:18528. doi: 10.1038/s41598-020-74485-z (PMC7595089; doi:10.1038/s41598-020-74485-z)
Supplement: Supplementary file 1 — Supplementary Figure Legends. [file 41598_2020_74485_MOESM1_ESM.docx]

**Supplemental Figure Legends**

**Fig. S1.** Localization of *S. mansoni* fucosyltransferases (SmFucTs). GFP-tagged SmFucTs were co-expressed with three different RFP-tagged Golgi reference markers (GnTI, XylT and ST) in *N. benthamiana* leaves. The ‘donut-shaped Golgi body’ and the ‘side-on view Golgi body’ were observed for all SmFucT. As an example, merged representative pictures are given for SmFucTE in green with the three different reference markers in violet, whereas co-localization is seen in white. The boxed areas represent a magnification of one side-on view Golgi body used for Pearson’s correlation coefficient analysis. The scale bar equals 1 μm.

**Fig. S2.** Sub-Golgi localization if *S. mansoni* fucosyltransferases (SmFucTs). GFP-tagged SmFucTs and RFP-tagged Golgi markers GnTI, XylT and ST were co-expressed in *N. benthamiana* leaves and co-localization was analyzed three days post infiltration with confocal microscopy. Merged representative pictures of each SmFucT (in green) with the three reference markers (in violet) are depicted. Co-localization is seen in white. The scale bar indicates 1 μm.

**Fig. S3.** Core fucosylation of omega-1 N-glycans by fucosyltransferases of *S. mansoni* (SmFucTs). Omega-1 was co-expressed in ΔXT/FT *N. benthamiana* plants with SmFucTs (F, J, K or L). After extraction and subsequent purification from the apopolast fluid the glycan composition of omega-1 was analyzed my MALDI-TOF-MS. MS profiles are given for omega-1 released N-glycans upon co-expression of SmFucTF (A), SmFucTJ (B), SmFucTK (C) and SmFucTL (D).

**Fig. S4.** Confirmation of the synthesis of Lewis X by enzymatic digestion. MALDI-TOF MS profiles of kappa-5 N-glycans upon Lewis X engineering with SmFucTD (A), SmFucTE (B) or SmFucTF (C). Profiles are given of N-glycans before and after digestion with β1,4/6-galactosidase and α1,3/4-fucosidase for which the substrate specificity is indicated in the dashed box. This enzymatic digest confirms the presence of Lewis X.

**Fig. S5.** Confirmation of the synthesis of Lewis X by enzymatic digestion. MALDI-TOF MS profiles of kappa-5 N-glycans upon Lewis X engineering with SmFucTD (A), SmFucTE (B) or SmFucTF (C). Profiles are given of N-glycans before and after digestion with β1,3-galactosidase and α1,3/4-fucosidase for which the substrate specificity is indicated in the dashed box. This enzymatic digest confirms the absence of Lewis A, which could occur on plant glycoproteins.

**Fig. S6.** Synthesis of LeX on kappa-5 N-glycans by fucosyltransferases of *S. mansoni* (SmFucTs). Kappa-5 was co-expressed in ΔXT/FT *N. benthamiana* plants with sialDrGalT (GalT) and SmFucTA. After extraction of apoplast proteins the N-glycan composition was analyzed by MALDI-TOF MS. MS profiles are given for kappa-5 released N-glycans before (A) and after enzymatic treatment with β1,4/6-galactosidase and α1,3/4-fucosidase (B) or β1,3-galactosidase and α1,3/4-fucosidase (C).

**Fig. S7.** Confirmation of LDN-F and F-LDN synthesis by enzymatic digestions. MALDI-TOF MS profiles of kappa-5 N-glycans upon engineering of fucosylated LDN with sialTnFucT9a (A), SmFucTD (B), SmFucTE (C) or SmFucTF (D). Profiles are given of N-glycans before and after digestion with a combination of β-*N*-acetyl-glucosaminidase and β-*N*-acetyl-hexosaminidase for which the substrate specificities are indicated in the dashed boxes. Monosaccharides for which the positions are not clear (prior to enzymatic digestion) are indicated between brackets.

**Fig. S8.** Confirmation of the synthesis of F-LDN-F by enzymatic digestion. MALDI-TOF MS profiles for kappa-5 N-glycans upon engineering F-LDN-F with SmFucTF plus SmFucTD (A) or SmFucTE (B). Profiles are given of N-glycans before and after digestion with a combination of β-*N*-acetyl-glucosaminidase and β-*N*-acetyl-hexosaminidase for which the substrate specificities are indicated in the dashed boxes. When a MS peak represents multiple N-glycan structures of identical mass, the number of monosaccharide residues for which the position on the N-glycan is not clear is indicated above the glycan and the possible positions of these residues are indicated between brackets. It is assumed that SmFucTD and SmFucTE synthesize LDN-F as efficient as when they are expressed alone (see Fig. S7).
