# Supplementary material for: Functional characterization of Schistosoma mansoni fucosyltransferases in Nicotiana benthamiana plants
Source: Sci Rep. 2020 Oct 28;10:18528. doi: 10.1038/s41598-020-74485-z (PMC7595089; doi:10.1038/s41598-020-74485-z)
Supplement: Supplementary file 2 — Supplementary Information. [file 41598_2020_74485_MOESM2_ESM.pdf]

Table S1. Putative *Schistosoma mansoni* fucosyltransferases (SmFucTs).
This table describes the putative SmFucT coding sequences reported in literature and found in the databases UniProtKB and GeneDB. The transmembrane domain (TMD) was predicted by the TMHMM server v2.0. Based on these predictions the length of the cytoplasmic tail (CT) and the TMD were calculated (indicated in amino acids (aa)). X indicates that no sequence was found or no TMD was predicted by the TMHMM server v2.0. SmFucTA to M, amplified from cDNA by Peterson and colleagues[20], were used as reference sequences. The percentage of identity between the putative SmFucT coding amino acid (aa) sequences and the most similar protein reference sequences were determined by Clustal Omega alignment. If the percentage of identity exceeded 70% differences are indicated according to the protein mutation nomenclature of den Dunnen and Antonarakis[48]. The differences between the SmFucTs amplified from cDNA are only indicated when the percentage of identity exceeded 90%.

Protein	Length (aa)	TMD (length CT/TMD in aa)	Percentage of identity (highest)	Sequence diversity and sequence related information	Published in
SmFucTA	426aa	20-37 (19/18)	47% with SmFucTD		20, 21, 49, 50, 51
smp_148850	x			on ncbi linked with SmFucTA described by Trottein and colleagues [2]	20, 25, 26
smp_211180	643aa	x	92.88% with SmFucTA	p.M1_K61del, p.K62M, p.N398Mfs*308	51, 52
smp_214370.1	426aa	20-37 (19/18)	100% with SmFucTA		50
smp_129730	49aa	x	30.43% with SmFucTA		51
smp_214380.1	338aa	x	21.55% with SmFucTA		50
SmFucTB	416aa	7-29 (6/23)	50.72% with SmFucTF		20, 21, 50, 51
smp_099090	347aa	x	21.09% with SmFucTB		20, 25, 26, 51
smp_209060	277aa	7-29 (6/23)	99.28% with SmFucTB	previously smp_109500, p.V162I, p.I181_E319del	50, 51, 52
A0A3Q0KJK9	416aa	7-29 (6/23)	98.08% with SmFucTB	p.V162I, p.V156E, p.P157A, p.K170S, p.T263I, p.S306G, p.A316V, p.C320G	51
A0A146MI48	416aa	7-29 (6/23)	97.6% with SmFucTB	p.Y48H, p.V162I, p.V166E, p.P167A, p.K170S, p.I254V, p.G378E, p.S306G, p.A316V, p.E320G	51
smp_109500	x			see smp_209060	26
SmFucTC	463aa	13-35 (12/23)	41.38% with SmFucTA		20, 21, 51
smp_154410	311aa	x	100% with SmFucTC	p.M1_L152del	21, 25, 50, 51
A0A3Q0KQD9	148aa	13-35 (12/23)	97.3% with SmFucTC	p.N148Lfs*2	51

Protein	Length (aa)	TMD (length CT/TMD in aa)	Percentage of identity	Sequence diversity and sequence related information	Published in
SmFucTD	398aa	13-35 (12/23)	47% with SmFucTA		20, 21, 50, 51
smp_054300	394aa	13-35 (12/23)	100% with SmFucTD	p.N395*	20, 25, 26, 50, 51, 52, 53
A0A3Q0KG08	324aa	x	99.69% with SmFucTD	p.M1_V74del, p.I346K	51
smp_129750	304aa	x	50.99% with SmFucTD		20, 25, 50, 51, 52
SmFucTE	420aa	17-39 (16/23)	69.63% with SmFucTF		20, 21, 50, 51, 52
smp_028910	101aa	x	100% with SmFucTE	previously smp_205640, p.M1_V326del	50, 51, 52
smp_137740	271aa	17-39 (16/23)	94.46% with SmFucTE	p.V190_K355delins15, p.N420K, p.G421D, p.R422H	20, 25, 25, 50, 51, 52
smp_205640	x			see smp_028910	50, 51, 52
A0A3Q0KMV7	355aa	17-39 (16/23)	98.59% with SmFucTE	p.Y44H, p.V190-S262del, p.N420K, p.G421D, p.R422H	51
SmFucTF	434aa	21-43 (20/23)	69.63% with SmFucTE		20, 21, 50, 51
smp_137730	31aa	x	93.55% with SmFucTF	p.M1_K295del, p.T317A, p.N326Kfs*2	20, 25, 26, 50, 51, 52, 53
smp_142860	139aa	x	100% with SmFucTF	p.M1_K295del	25, 26, 50, 51, 52
A0A3Q0KNN7	434aa	21-43 (20/23)	99.77% with SmFucTF	p.V36I	51
smp_193620	92aa	x	72.83% with SmFucTF	p.M1_P131del, p.I196Vfs*29	52
smp_193870	101aa	x	98.02% with SmFucTF	p.M1_V333del, p.Y348H, p.P395L	50, 51, 52
smp_194990	118aa	21-43 (20/23)	96.61% with SmFucTF	p.S25Q, p.V36I, p.H91Q, p.K118Sfs*2	20, 26, 50, 51, 52
SmFucTG	x			pseudogene	20, 50
SmFucTH	599aa	9-31 (8/23)	33.87% with SmFucTJ and SmFucTM		20, 50, 51
smp_175120.1	882aa	21-43 (20/23)	83.6% with SmFucTH	p.M1YextM-13, p.V3L, p.T4I, p.S5G, p.K7E, p.V31Nfs390, p.V31_G481ins379	20, 25, 50, 52

Protein	Length (aa)	TMD (length CT/TMD in aa)	Percentage of identity	Sequence diversity and sequence related information	Published in
SmFucTI	592aa	x	98.99% with SmFucTJ	p.G398R, p.L399S, p.S405R, p.E502K, p.G547R, p.K581T	[20, 50, 51]
SmFucTJ	592aa	x	98.99% with SmFucTI	p.R398G, p.S399L, p.R405S, p.K502E, p.R547G, p.T581K	[20, 50, 51]
smp_138730	474aa	x	93.88% with SmFucTJ	p.M1_S77del, p.K79R, p.T80L, p.T81M, p.R82I, p.N83D, p.V84G, p.Q85A, p.I86E, p.T87V, p.N88D, p.G258_S260del	[20, 25, 26, 50, 51, 52]
SmFucTK	579aa	12-30 (11/19)	87.65% with SmFucTL		[20, 50, 51]
smp_138750	502aa	x	100% with SmFucTK	p.M1_S77del	[20, 25, 50, 51, 52]
A0A3Q0KMV6	579aa	12-30 (11/19)	97.06% with SmFucTK	p.T80I, p.Q85K, p.E89V, p.S101R, p.L127F, p.Y157H, p.G146D, p.K175R, p.M176I, p.N180D, p.E187Q, p.N296D, p.Y309F, p.A318S, p.Q378R, p.F383L, p.T557A	[51]
SmFucTL	588aa	7-26 (6/20)	87.65% with SmFucTK		[20, 50, 51]
smp_030650	484aa	x	94.21% with SmFucTL	p.M1_K82del, p.I339M, p.I355_R406delin30, p.M452I	[50, 51]
SmFucTM	592aa	x	98.14% with SmFucTJ	p.S35C, p.S50P, p.L118S, p.I122L, p.N281H, p.H283L, p.R324K, p.E336K, p.V342G, p. G398R, p.L399S	[20, 51]
smp_212520	147aa	x	97.96% with SmFucTM	p.M1_K200del p.V345Cfs*4	[50, 51, 52]
smp_185720	x				[25, 26, 53]
smp_189280	x				[25]
smp_138740	x			found on same scaffold as SmFucTJ, K and L, but could not be amplified by Peterson and colleagues [1]	[20, 25]

Additional references:

48. den Dunnen, J. T. & Antonarakis, S. E. Nomenclature for the description of human sequence variations. *Hum. Genet.* ***109***, 121-124 (2001).

49. Trottein, F. *et al.* Molecular cloning of a putative α3-fucosyltransferase from Schistosoma mansoni. *Molecular and Biochemical Parasitology* vol. 107 279–287 (2000).

50. Logan-Klumpler, F. J. *et al.* GeneDB-an annotation database for pathogens. *Nucleic Acids Res.* **40**, 98–108 (2012).

51. Bateman, A. UniProt: A worldwide hub of protein knowledge. *Nucleic Acids Res.* **47**, D506–D515 (2019).

52. Mickum, M. L., Prasanphanich, N. S., Heimburg-Molinaro, J., Leon, K. E. & Cummings, R. D. Deciphering the glycogenome of schistosomes. *Front. Genet.* **5**, 1–15 (2014).

53. Parker-Manuel, S. J., Ivens, A. C., Dillon, G. P. & Wilson, R. A. Gene expression patterns in larval Schistosoma mansoni associated with infection of the mammalian host. *PLoS Negl. Trop. Dis.* **5**, (2011).
