# Supplementary material for: Publisher Correction: Functional characterization of Schistosoma mansoni fucosyltransferases in Nicotiana benthamiana plants
Source: Sci Rep. 2021 Feb 17;11:4368. doi: 10.1038/s41598-021-83766-0 (PMC7889881; doi:10.1038/s41598-021-83766-0)
Supplement: Supplementary file 1 — Supplementary Information. [file 41598_2021_83766_MOESM1_ESM.pdf]

Supplementary information belonging to the manuscript:

Functional characterization of *Schistosoma mansoni* fucosyltransferases in *Nicotiana benthamiana* plants

Kim van Noort[1], Dieu-Linh Nguyen[2], Verena Kriechbaumer[3], Chris Hawes[3], Cornelis H. Hokke[2], Arjen Schots[1] and Ruud H. P. Wilbers[1]*

1 Laboratory of Nematology, Plant Sciences Group, Wageningen University and Research, Droevendaalsesteeg 1, 6708 PB, Wageningen, The Netherlands

2 Department of Parasitology, Leiden University Medical Center, Albinusdreef 2333 ZA, Leiden, The Netherlands

3 Department of Biological and Medical Sciences, Oxford Brookes University, Oxford OX3 0BP, United Kingdom

*Corresponding author:

Ruud H.P. Wilbers, P.O. Box 8123, 6700 ES Wageningen, The Netherlands, phone: +31(0)317485261, fax: +31(0)317484254, e-mail: ruud.wilbers@wur.nl

Table S1. Putative *Schistosoma mansoni* fucosyltransferases (SmFucTs).
This table describes the putative SmFucT coding sequences reported in literature and found in the databases UniProtKB and GeneDB. The transmembrane domain (TMD) was predicted by the TMHMM server v2.0. Based on these predictions the length of the cytoplasmic tail (CT) and the TMD were calculated (indicated in amino acids (aa)). X indicates that no sequence was found or no TMD was predicted by the TMHMM server v2.0. SmFucTA to M, amplified from cDNA by Peterson and colleagues[20], were used as reference sequences. The percentage of identity between the putative SmFucT coding amino acid (aa) sequences and the most similar protein reference sequences were determined by Clustal Omega alignment. If the percentage of identity exceeded 70% differences are indicated according to the protein mutation nomenclature of den Dunnen and Antonarakis[47]. The differences between the SmFucTs amplified from cDNA are only indicated when the percentage of identity exceeded 90%.

Protein	Length (aa)	TMD (length CT/TMD in aa)	Percentage of identity (highest)	Sequence diversity and sequence related information	Published in
SmFucTA	426aa	20-37 (19/18)	47% with SmFucTD		20, 21, 49, 50, 51
smp_148850	x			on ncbi linked with SmFucTA described by Trottein and colleagues [2]	20, 25, 26
smp_211180	643aa	x	92.88% with SmFucTA	p.M1_K61del, p.K62M, p.N398Mfs*308	51, 52
smp_214370.1	426aa	20-37 (19/18)	100% with SmFucTA		50
smp_129730	49aa	x	30.43% with SmFucTA		51
smp_214380.1	338aa	x	21.55% with SmFucTA		50
SmFucTB	416aa	7-29 (6/23)	50.72% with SmFucTF		20, 21, 50, 51
smp_099090	347aa	x	21.09% with SmFucTB		20, 25, 26, 51
smp_209060	277aa	7-29 (6/23)	99.28% with SmFucTB	previously smp_109500, p.V162I, p.I181_E319del	50, 51, 52
A0A3Q0KJK9	416aa	7-29 (6/23)	98.08% with SmFucTB	p.V162I, p.V156E, p.P157A, p.K170S, p.T263I, p.S306G, p.A316V, p.C320G	51
A0A146MI48	416aa	7-29 (6/23)	97.6% with SmFucTB	p.Y48H, p.V162I, p.V166E, p.P167A, p.K170S, p.I254V, p.G378E, p.S306G, p.A316V, p.E320G	51
smp_109500	x			see smp_209060	26
SmFucTC	463aa	13-35 (12/23)	41.38% with SmFucTA		20, 21, 51
smp_154410	311aa	x	100% with SmFucTC	p.M1_L152del	21, 25, 50, 51
A0A3Q0KQD9	148aa	13-35 (12/23)	97.3% with SmFucTC	p.N148Lfs*2	51

Protein	Length (aa)	TMD (length CT/TMD in aa)	Percentage of identity	Sequence diversity and sequence related information	Published in
SmFucTD	398aa	13-35 (12/23)	47% with SmFucTA		20, 21, 50, 51
smp_054300	394aa	13-35 (12/23)	100% with SmFucTD	p.N395*	20, 25, 26, 50, 51, 52, 53
A0A3Q0KG08	324aa	x	99.69% with SmFucTD	p.M1_V74del, p.I346K	51
smp_129750	304aa	x	50.99% with SmFucTD		20, 25, 50, 51, 52
SmFucTE	420aa	17-39 (16/23)	69.63% with SmFucTF		20, 21, 50, 51, 52
smp_028910	101aa	x	100% with SmFucTE	previously smp_205640, p.M1_V326del	50, 51, 52
smp_137740	271aa	17-39 (16/23)	94.46% with SmFucTE	p.V190_K355delins15, p.N420K, p.G421D, p.R422H	20, 25, 25, 50, 51, 52
smp_205640	x			see smp_028910	50, 51, 52
A0A3Q0KMV7	355aa	17-39 (16/23)	98.59% with SmFucTE	p.Y44H, p.V190-S262del, p.N420K, p.G421D, p.R422H	51
SmFucTF	434aa	21-43 (20/23)	69.63% with SmFucTE		20, 21, 50, 51
smp_137730	31aa	x	93.55% with SmFucTF	p.M1_K295del, p.T317A, p.N326Kfs*2	20, 25, 26, 50, 51, 52, 53
smp_142860	139aa	x	100% with SmFucTF	p.M1_K295del	25, 26, 50, 51, 52
A0A3Q0KNN7	434aa	21-43 (20/23)	99.77% with SmFucTF	p.V36I	51
smp_193620	92aa	x	72.83% with SmFucTF	p.M1_P131del, p.I196Vfs*29	52
smp_193870	101aa	x	98.02% with SmFucTF	p.M1_V333del, p.Y348H, p.P395L	50, 51, 52
smp_194990	118aa	21-43 (20/23)	96.61% with SmFucTF	p.S25Q, p.V36I, p.H91Q, p.K118Sfs*2	20, 26, 50, 51, 52
SmFucTG	x			pseudogene	20, 50
SmFucTH	599aa	9-31 (8/23)	33.87% with SmFucTJ and SmFucTM		20, 50, 51
smp_175120.1	882aa	21-43 (20/23)	83.6% with SmFucTH	p.M1YextM-13, p.V3L, p.T4I, p.S5G, p.K7E, p.V31Nfs390, p.V31_G481ins379	20, 25, 50, 52

Protein	Length (aa)	TMD (length CT/TMD in aa)	Percentage of identity	Sequence diversity and sequence related information	Published in
SmFucTI	592aa	x	98.99% with SmFucTJ	p.G398R, p.L399S, p.S405R, p.E502K, p.G547R, p.K581T	[20, 50, 51]
SmFucTJ	592aa	x	98.99% with SmFucTI	p.R398G, p.S399L, p.R405S, p.K502E, p.R547G, p.T581K	[20, 50, 51]
smp_138730	474aa	x	93.88% with SmFucTJ	p.M1_S77del, p.K79R, p.T80L, p.T81M, p.R82I, p.N83D, p.V84G, p.Q85A, p.I86E, p.T87V, p.N88D, p.G258_S260del	[20, 25, 26, 50, 51, 52]
SmFucTK	579aa	12-30 (11/19)	87.65% with SmFucTL		[20, 50, 51]
smp_138750	502aa	x	100% with SmFucTK	p.M1_S77del	[20, 25, 50, 51, 52]
A0A3Q0KMV6	579aa	12-30 (11/19)	97.06% with SmFucTK	p.T80I, p.Q85K, p.E89V, p.S101R, p.L127F, p.Y157H, p.G146D, p.K175R, p.M176I, p.N180D, p.E187Q, p.N296D, p.Y309F, p.A318S, p.Q378R, p.F383L, p.T557A	[51]
SmFucTL	588aa	7-26 (6/20)	87.65% with SmFucTK		[20, 50, 51]
smp_030650	484aa	x	94.21% with SmFucTL	p.M1_K82del, p.I339M, p.I355_R406delin30, p.M452I	[50, 51]
SmFucTM	592aa	x	98.14% with SmFucTJ	p.S35C, p.S50P, p.L118S, p.I122L, p.N281H, p.H283L, p.R324K, p.E336K, p.V342G, p. G398R, p.L399S	[20, 51]
smp_212520	147aa	x	97.96% with SmFucTM	p.M1_K200del p.V345Cfs*4	[50, 51, 52]
smp_185720	x				[25, 26, 53]
smp_189280	x				[25]
smp_138740	x			found on same scaffold as SmFucTJ, K and L, but could not be amplified by Peterson and colleagues [1]	[20, 25]

Table S2. Expression profiles of *Schistosoma mansoni* fucosyltransferases (SmFucTs).

Expression of SmFucT genes throughout different life stages was obtained by meta-analysis of published RNAseq studies (Lu *et al.* 2018; https://doi.org/10.1101/308213). For each SmFucT gene the life stages are indicated in which highest expression levels are found. For each SmFucT a link is provided to the complete expression profile.

Protein	Most highly expressed life stage (*normalized expression level*)	Expression profile (link)
SmFucTA		
smp_214370	Adults (male: *80.26*; female: *49.66*)	https://meta.schisto.xyz/smansoni/smp_214370/
smp_214380	Gonads (male: *210.30*; female *75.49*)	https://meta.schisto.xyz/smansoni/smp_214380/
SmFucTB		
smp_209060	Gonads (male: *21.32*; female *23.20*)	https://meta.schisto.xyz/smansoni/smp_209060/
SmFucTC		
smp_154410	Cercariae (*18.38*) and schistosomula (*18.76*)	https://meta.schisto.xyz/smansoni/smp_154410/
SmFucTD		
smp_054300	Miracidium (*51.31*) and sporocysts (*40.48*)	https://meta.schisto.xyz/smansoni/smp_054300/
smp_129750	Female gonads (*0.47*) and schistosomula (*0.40*)	https://meta.schisto.xyz/smansoni/smp_129750/
SmFucTE		
smp_028910	Male gonads (*21.94*)	https://meta.schisto.xyz/smansoni/smp_028910/
smp_137740	Male gonads (*43.45*) and eggs (*25.10*)	https://meta.schisto.xyz/smansoni/smp_137740/
smp_205640	Male gonads (*70.65*)	https://meta.schisto.xyz/smansoni/smp_205640/
SmFucTF		
smp_137730	Male gonads (*24.48*)	https://meta.schisto.xyz/smansoni/smp_137730/
smp_142860	Male gonads (*20.26*) and eggs (*16.04*)	https://meta.schisto.xyz/smansoni/smp_142860/
smp_193870	Female gonads (*1.41*)	https://meta.schisto.xyz/smansoni/smp_193870/
smp_194990	Eggs (*29.32*) new name: smp_243310<br>Male gonads (*8.31*) new name: smp_199790	https://meta.schisto.xyz/smansoni/smp_243310/<br>https://meta.schisto.xyz/smansoni/smp_199790/
SmFucTH		
smp_175120	Adults (*25.84*), schistosomula (*16.37*) and cercariae (*12.89*)	https://meta.schisto.xyz/smansoni/smp_175120/
SmFucTJ		
smp_138730	Sporocysts (*11.50*) and females (*12.04*)	https://meta.schisto.xyz/smansoni/smp_138730/
SmFucTK		
smp_138750	Male gonads (*34.25*) and males (*27.80*)	https://meta.schisto.xyz/smansoni/smp_138750/
SmFucTL		
smp_030650	Male gonads (*6.71*)	https://meta.schisto.xyz/smansoni/smp_030650/
SmFucTM		
smp_212520	Females (*19.31*)	https://meta.schisto.xyz/smansoni/smp_212520/

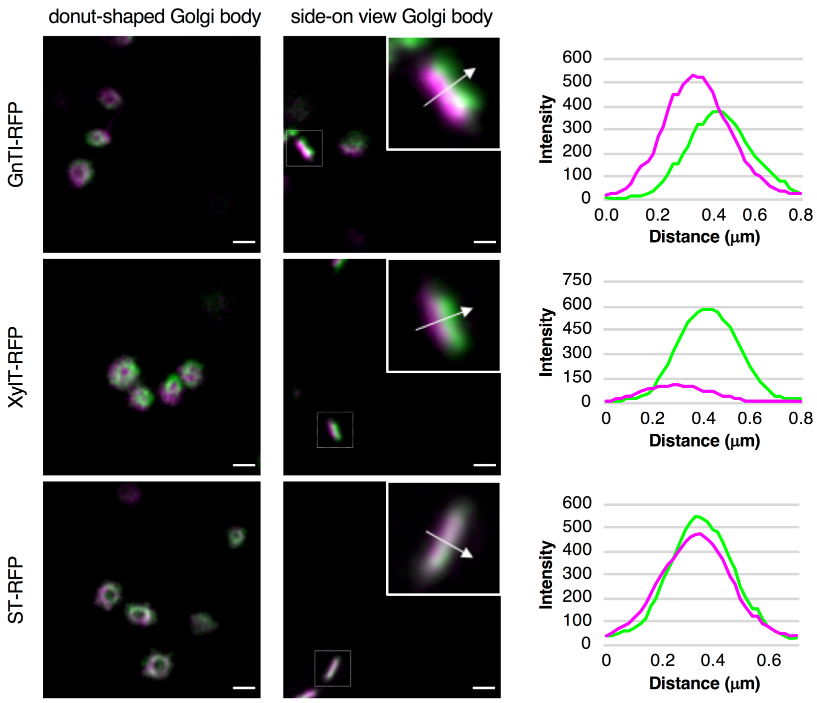

Fig. S1. Localization of *S. mansoni* fucosyltransferases (SmFucTs). GFP-tagged SmFucTs were co-expressed with three different RFP-tagged Golgi reference markers (GnTI, XylT and ST) in *N. benthamiana* leaves. The 'donut-shaped Golgi body' and the 'side-on view Golgi body' were observed for all SmFucT. As an example, merged representative pictures are given for SmFucTE in green with the three different reference markers in violet, whereas co-localization is seen in white. The boxed areas represent a magnification of one side-on view Golgi body used for Pearson's correlation coefficient analysis. The scale bar equals 1 µm.

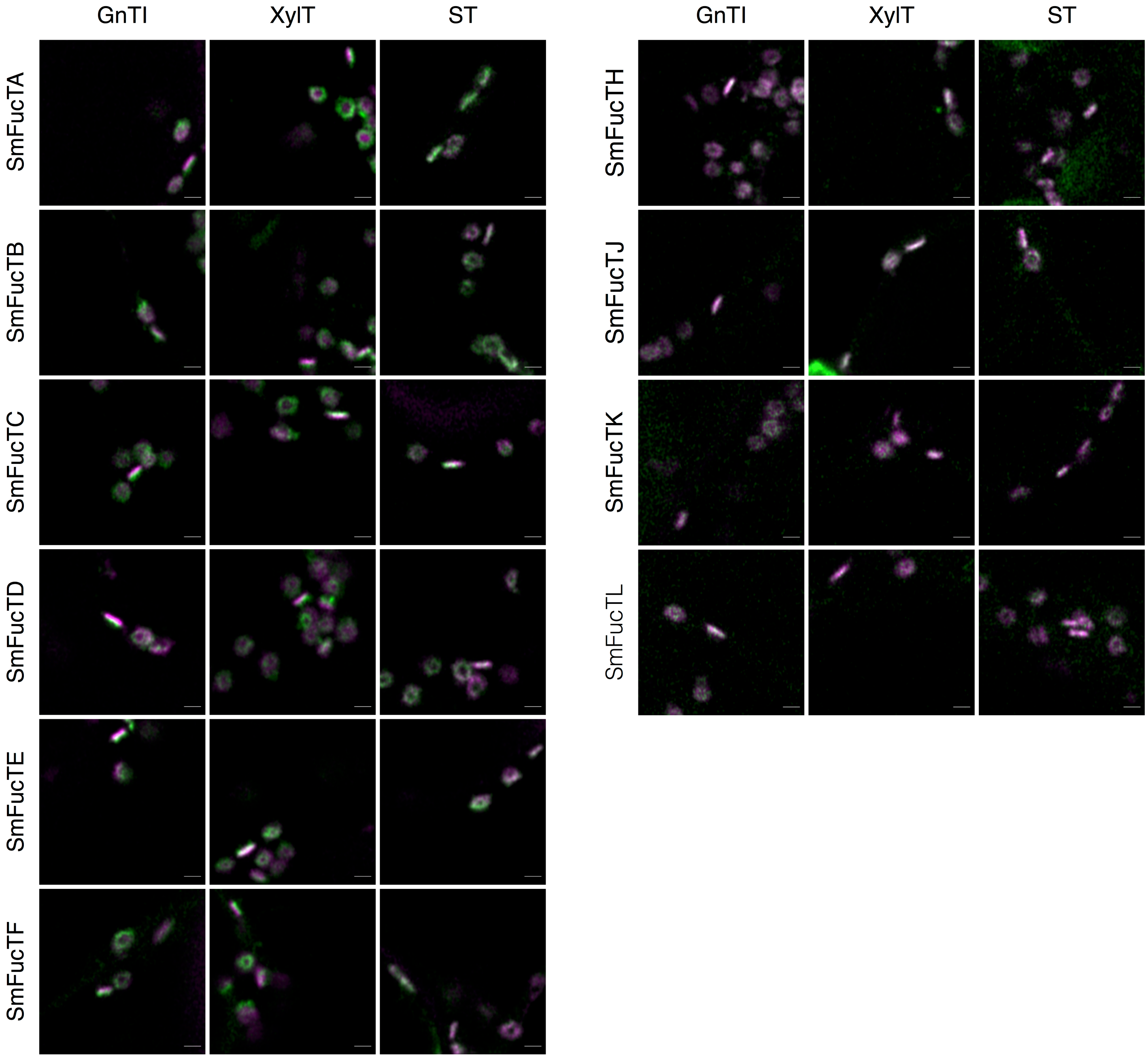

Fig. S2. Sub-Golgi localization if *S. mansoni* fucosyltransferases (SmFucTs). GFP-tagged SmFucTs and RFP-tagged Golgi markers GnTI, XylT and ST were co-expressed in *N. benthamiana* leaves and co-localization was analyzed three days post infiltration with confocal microscopy. Merged representative pictures of each SmFucT (in green) with the three reference markers (in violet) are depicted. Co-localization is seen in white. The scale bar indicates 1 µm.

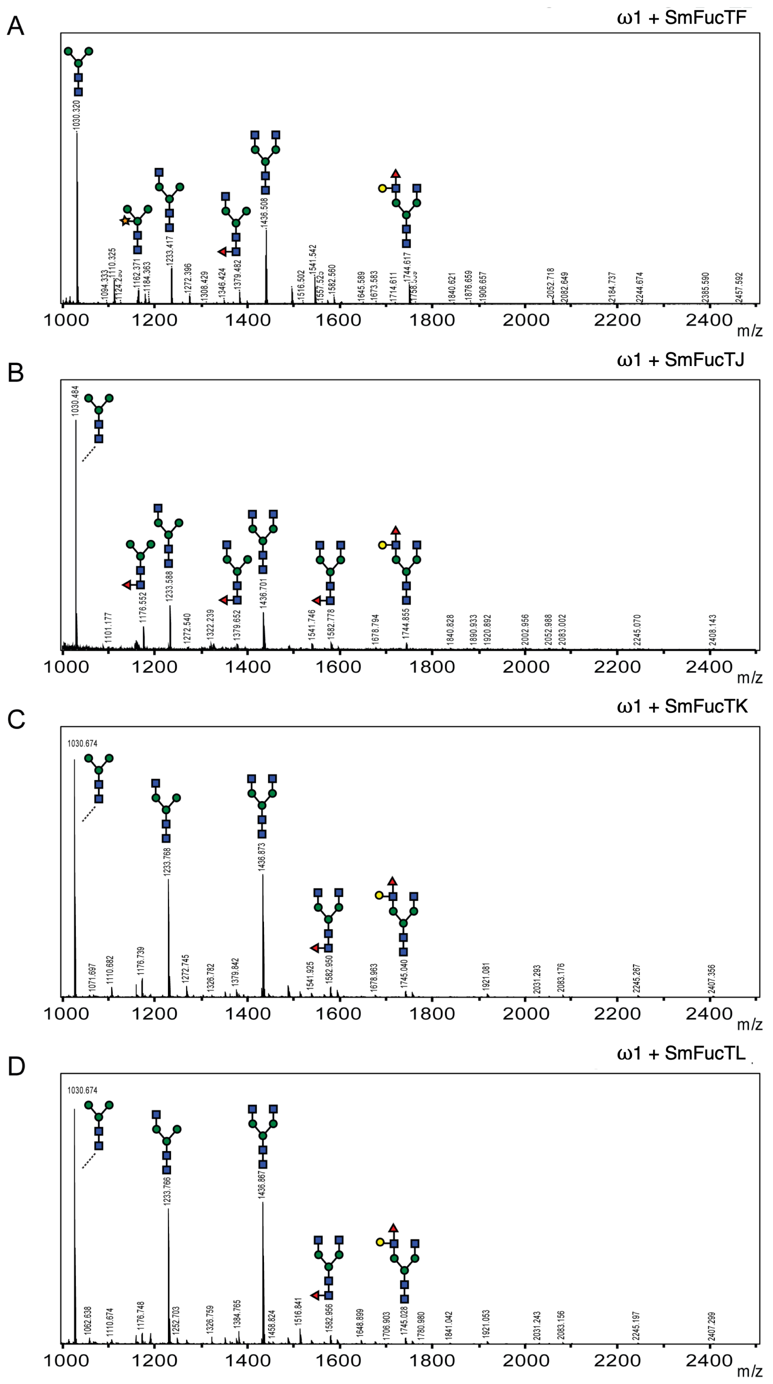

Fig. S3. Core fucosylation of omega-1 N-glycans by fucosyltransferases of *S. mansoni* (SmFucTs). Omega-1 was co-expressed in ΔXT/FT *N. benthamiana* plants with SmFucTs (F, J, K or L). After extraction and subsequent purification from the apopolast fluid the glycan composition of omega-1 was analyzed my MALDI-TOF-MS. MS profiles are given for omega-1 released N-glycans upon co- expression of SmFucTF (A), SmFucTJ (B), SmFucTK (C) and SmFucTL (D).

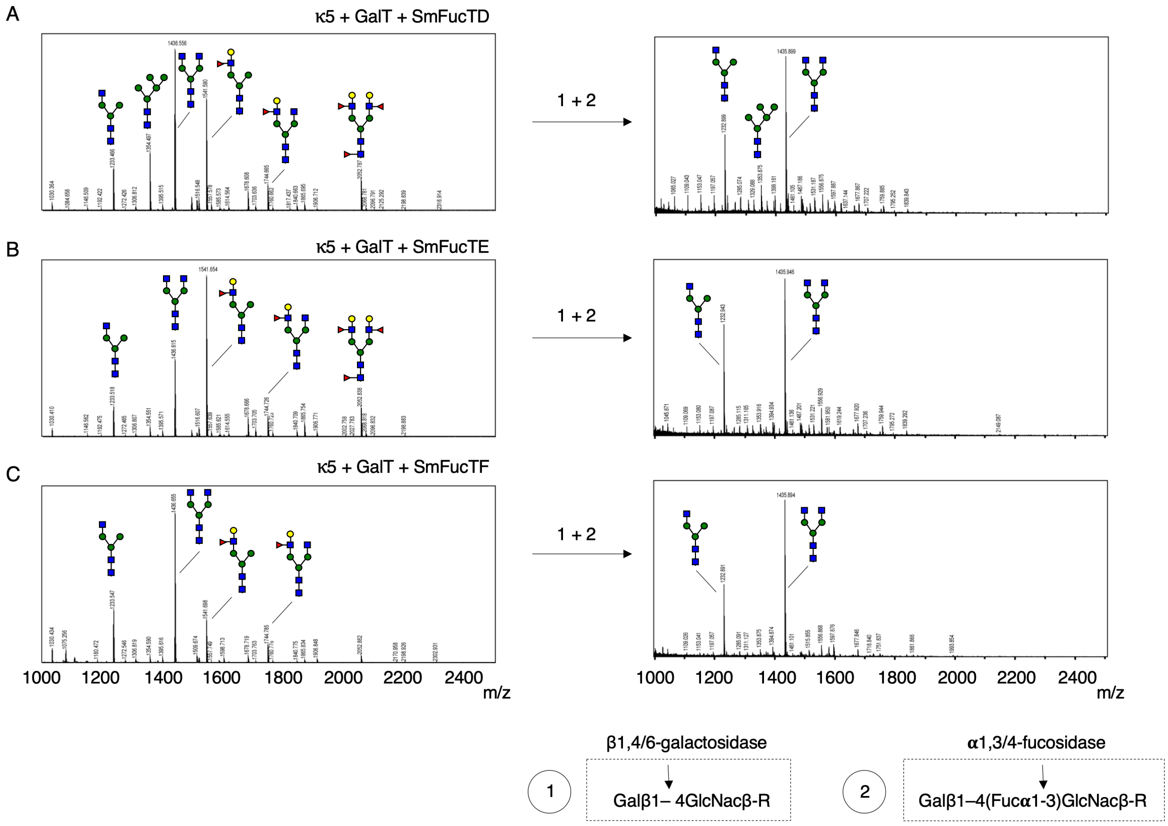

Fig. S4. Confirmation of the synthesis of Lewis X by enzymatic digestion. MALDI-TOF MS profiles of kappa-5 N-glycans upon Lewis X engineering with SmFucTD (A), SmFucTE (B) or SmFucTF (C). Profiles are given of N-glycans before and after digestion with β1,4/6-galactosidase and α1,3/4- fucosidase for which the substrate specificity is indicated in the dashed box. This enzymatic digest confirms the presence of Lewis X.

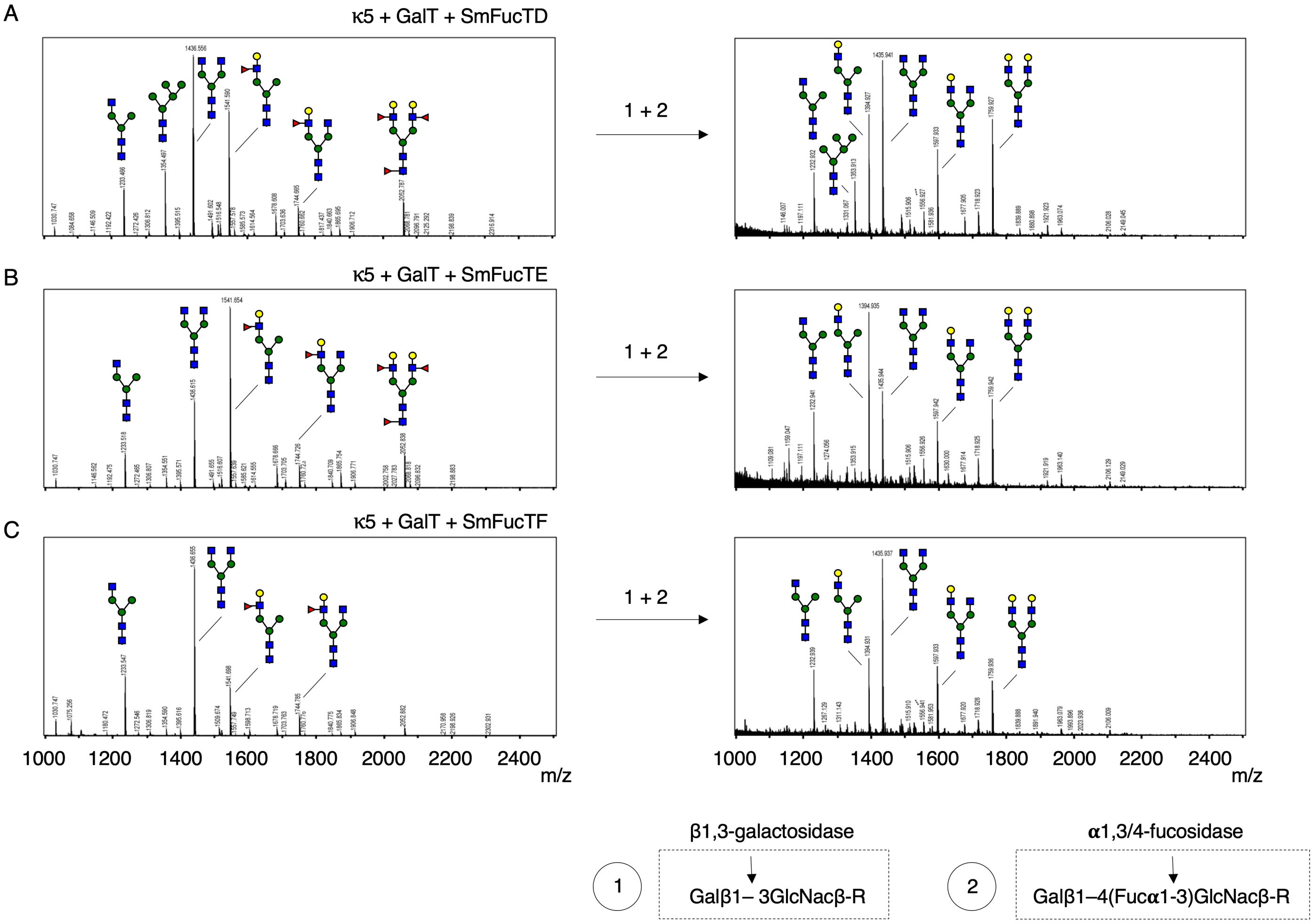

Fig. S5. Confirmation of the synthesis of Lewis X by enzymatic digestion. MALDI-TOF MS profiles of kappa-5 N-glycans upon Lewis X engineering with SmFucTD (A), SmFucTE (B) or SmFucTF (C). Profiles are given of N-glycans before and after digestion with β1,3-galactosidase and α1,3/4- fucosidase for which the substrate specificity is indicated in the dashed box. This enzymatic digest confirms the absence of Lewis A, which could occur on plant glycoproteins.

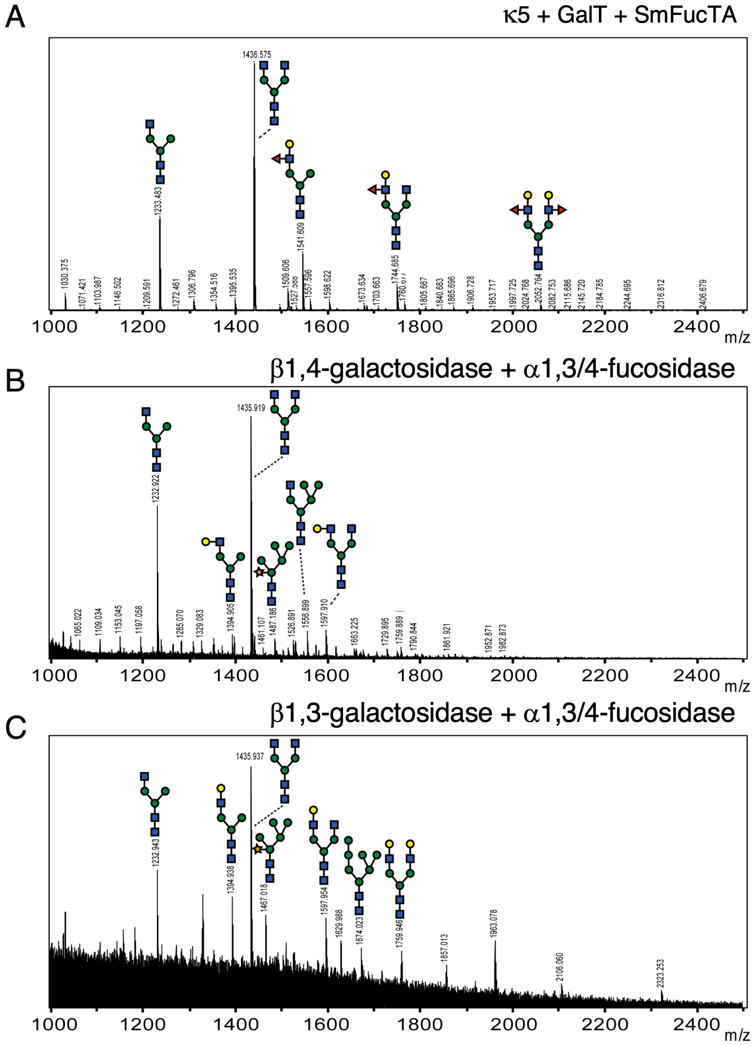

Fig. S6. Synthesis of LeX on kappa-5 N-glycans by fucosyltransferases of *S. mansoni* (SmFucTs). Kappa-5 was co-expressed in ΔXT/FT *N. benthamiana* plants with sialDrGalT (GalT) and SmFucTA. After extraction of apoplast proteins the N-glycan composition was analyzed by MALDI-TOF MS. MS profiles are given for kappa-5 released N-glycans before (A) and after enzymatic treatment with β1,4/6-galactosidase and α1,3/4-fucosidase (B) or β1,3-galactosidase and α1,3/4-fucosidase (C).

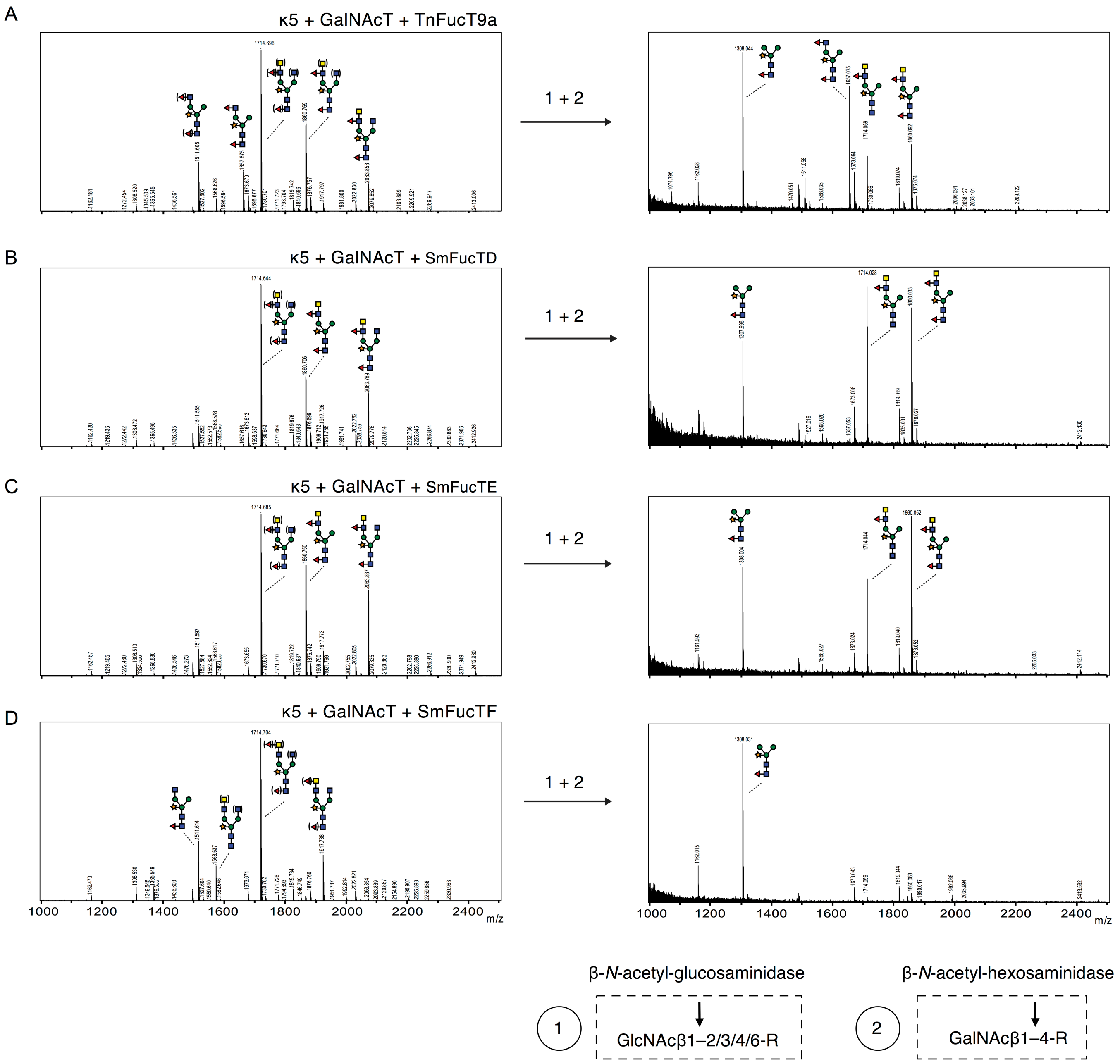

Fig. S7. Confirmation of LDN-F and F-LDN synthesis by enzymatic digestions. MALDI-TOF MS profiles of kappa-5 N-glycans upon engineering of fucosylated LDN with sialTnFucT9a (A), SmFucTD (B), SmFucTE (C) or SmFucTF (D). Profiles are given of N-glycans before and after digestion with a combination of β-*N*-acetyl-glucosaminidase and β-*N*-acetyl-hexosaminidase for which the substrate specificities are indicated in the dashed boxes. Monosaccharides for which the positions are not clear (prior to enzymatic digestion) are indicated between brackets.

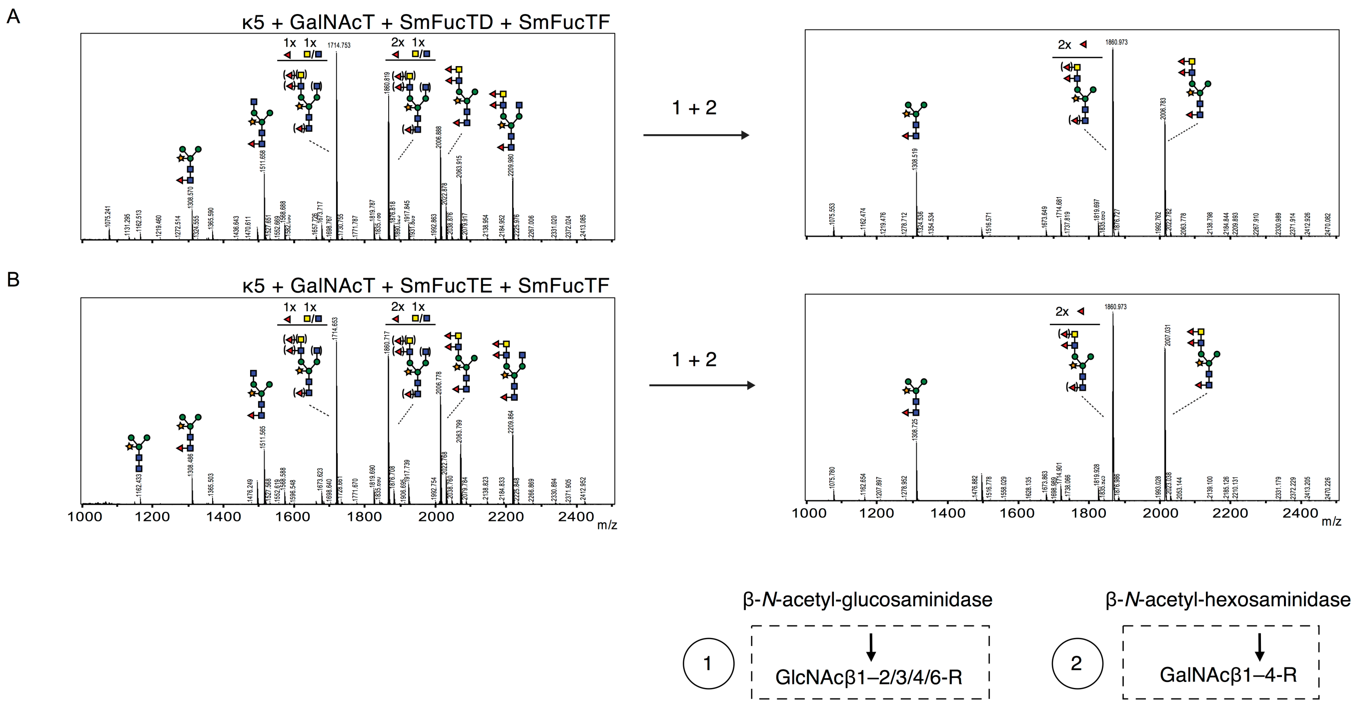

Fig. S8. Confirmation of the synthesis of F-LDN-F by enzymatic digestion. MALDI-TOF MS profiles for kappa-5 N-glycans upon engineering F-LDN-F with SmFucTF plus SmFucTD (A) or SmFucTE (B). Profiles are given of N-glycans before and after digestion with a combination of β-*N*-acetyl- glucosaminidase and β-*N*-acetyl-hexosaminidase for which the substrate specificities are indicated in the dashed boxes. When a MS peak represents multiple N-glycan structures of identical mass, the number of monosaccharide residues for which the position on the N-glycan is not clear is indicated above the glycan and the possible positions of these residues are indicated between brackets. It is assumed that SmFucTD and SmFucTE synthesize LDN-F as efficient as when they are expressed alone (see Fig. S7).
